# Abundance and biomass of copepods and cladocerans in Atlantic and Arctic domains of the Barents Sea ecosystem

**DOI:** 10.1093/plankt/fbad043

**Published:** 2023-10-12

**Authors:** Hein Rune Skjoldal, Johanna Myrseth Aarflot

**Affiliations:** Institute of Marine Research, Ecosystem Processes Research Group, PO Box 1870 Nordnes, N-5817 Bergen, Norway; Institute of Marine Research, Ecosystem Processes Research Group, PO Box 1870 Nordnes, N-5817 Bergen, Norway

**Keywords:** zooplankton biomass, Calanus, copepods, cladocerans, size fractions

## Abstract

Zooplankton in the Barents Sea have been monitored annually with a standard procedure with determination of size-fractioned biomass since the mid-1980s. Biomass of copepods and cladocerans was estimated based on measured abundance and individual weights taken from literature. *Calanus* species were dominant, making up ~85% of the estimated biomass of copepods. The second most important taxon was *Oithona* spp. (~0.5 g dry weight (dw) m^−2^, ~10%), followed by *Metridia* spp. (~0.15 g dw m^−2^, 2–3%) and *Pseudocalanus* spp. (0.10–0.15 g dw m^−2^, 1–5%). Estimated biomass of cladoceran taxa (*Evadne* and *Podon*) was low (0.01 g dw m^−2^). *Calanus* spp. contributed most of the biomass of the medium size fraction (1–2 mm), whereas small copepod species (*Oithona*, *Pseudocalanus* and others) contributed to the small size fraction (<1 mm). Estimated biomass of *Calanus* spp. and of the sum of small copepod species were both positively correlated with measured total zooplankton biomass (*R*^2^ = 0.72 and 0.34, respectively). The biomass ratio of small copepod species to *Calanus* was similar in Atlantic and Arctic water masses (~0.15–0.2) but tended to increase with decreasing total biomass. This suggests a shift to relatively larger roles of small copepods as *Calanus* and total biomass decrease.

## INTRODUCTION

Copepods are typically a dominant group of zooplankton both in terms of numbers and biomass, especially in cold waters at high latitudes (Longhurst, 1985; Brandão *et al.*, 2021). “Large” calanoid copepods of genera *Calanus* and *Neocalanus* are key species in northern boreal, subarctic and Arctic marine ecosystems in the North Atlantic and North Pacific (Conover, 1988). Their importance is related to the ability to survive the long winter in a non-feeding passive state. In the Barents Sea, the boreal *Calanus finmarchicus* and the Arctic *C. glacialis* are dominant species in the southern and northern regions, respectively (Melle and Skjoldal, 1998; Falk-Petersen *et al.*, 2009), where they make up ~70–80% of the zooplankton biomass (Aarflot *et al.*, 2018). In addition to “large” copepods (2–8-mm prosome length), small copepods ~0.5–1 mm also play important roles in marine ecosystems (Norrbin, 1991; Paffenhöfer, 1993; Turner, 2004).

Cladocerans are another group of small planktonic crustaceans. They are important in freshwater ecosystems, where their role relative to copepods is part of the “size efficiency hypothesis,” formulated by Brooks and Dodson (1965) and Hall *et al.* (1976). Cladocerans are less common in marine waters (Egloff *et al.*, 1997; Brandão *et al.*, 2021), although they can be important in estuarine environments such as the Baltic Sea (Möllmann *et al.*, 2002). They are found in the Norwegian Coastal Current (NCC), which originates from the Baltic Sea outflow. Cladocerans can be important in neritic coastal waters, and the species *Evadne nordmanni* dominated the zooplankton composition in a Norwegian fjord in a case study reported by Skjoldal *et al.* (2013). The NCC continues north into the Barents Sea (Skagseth *et al.*, 2011) and may potentially transport cladocerans into this ecosystem.

Zooplankton in the Barents Sea have been monitored with a standardized procedure since the mid-1980s by the Institute of Marine Research (IMR) in Norway on a broad-scale autumn survey (Eriksen *et al.*, 2018; Skjoldal, 2023). Each zooplankton sample is split into two halves: one for determination of dry weight biomass in three size fractions, while the other half is preserved for later taxonomic analysis (Skjoldal *et al.*, 2013, 2022). An extensive data set on size-fractioned zooplankton biomass from these surveys has been used to document changes in the Barents Sea ecosystem in relation to fish predation and climate variability and change (Stige *et al.*, 2014; Dalpadado *et al.*, 2020; Skjoldal *et al.*, 2022; Skjoldal, 2023). The biomass data are reported annually and used in ecosystem assessments by the ICES Working Group on Integrated Ecosystem Assessments of the Barents Sea (WGIBAR, 2021).

Since taxonomic analysis is labor intensive, only a small fraction of the samples collected annually are processed for species counts. Nevertheless, due to the length of the time series, a decent number of samples (>500) with both biomass estimation (in size fractions) and species counts from the same zooplankton haul have accumulated over the years. We have previously used these data to estimate the biomass of *Calanus* species and their contribution to total zooplankton biomass in the Barents Sea, found to be ~ 80% on average (Aarflot *et al.*, 2018). Here, we expand our focus to also estimate biomass of other copepod species and cladocerans with the same data set as Aarflot *et al.* (2018). We quantify the contribution to biomass by small copepods, such as *Oithona*, *Pseudocalanus* and *Microcalanus* species, relative to the “large” *Calanus* species, noting that *Calanus* are also small copepods when they occur as young copepodite stages (Tande, 1991). The biomass of copepods and cladocerans estimated from species counts are compared to measured biomass in the associated half-samples. The separation of copepods and cladocerans into the three size fractions has been shown to be strictly size dependent (Skjoldal, 2021). We use this information to estimate the contribution of biomass of copepods and cladocerans to each size fraction as well as total biomass, in different water masses with Atlantic, Arctic or “mixed” temperature characteristics.

We have two main objectives for this study. The first is to provide a quantitative description of the community of copepods and cladocerans in terms of biomass distribution among the species. This includes quantification of small copepods and a reassessment of the dominance of *Calanus* species in terms of biomass. The second objective is to provide more information on the contribution of species of copepods to the three size fractions used in the operational monitoring of the Barents Sea ecosystem as reported annually by the ICES WGIBAR. The data set spans approximately three decades (1983–2016), with data collected mainly in the summer and autumn seasons. We treat the data separately for the two main domains, the Atlantic and Arctic water masses, as well as the mixed water mass in the polar front region between them (Ingvaldsen *et al.*, 2021). We illustrate variability of estimated copepod biomass across the total data set and for the summer period separately. Specifically, we address whether there is a shift in the biomass ratio of small copepod species to *Calanus* associated with a decline in total zooplankton biomass, which has been suggested to be linked to predation and warming climate (Skjoldal *et al.*, 2022; Skjoldal, 2023).

## METHODS

Zooplankton have been sampled with vertical hauls with a WP2 net (0.25-m^2^ opening, 180-μm mesh size; Skjoldal *et al.*, 2019) from near the seafloor (~10 m) to the surface. The WP2 net has been operated without a flowmeter, and depth-integrated biomass or numbers are expressed as the content of a net sample scaled up to 1-m^2^ sea surface, assuming 100% filtration efficiency. Clogging of the WP2 net with 180-μm mesh size is usually not a problem in the Barents Sea. The WP2 net is designed to have good filtration performance with a ratio of filter area (mesh openings) to net mouth area of 6 (Skjoldal *et al.*, 2013). Using equations in (Smith *et al.*, 1968; see Sameoto *et al.*, 2000, page 70), the volume that can be filtered before filtration efficiency drops below 85% is ~150 m^3^ in “green” water and ~1000 m^3^ in “blue” water. “Green” in this case was turbid nearshore waters at San Pedro in California with median Secchi depth readings of 4.5 m. Concentrations of chlorophyll *a* are typically low in the Barents Sea, even during spring phytoplankton blooms, which tend to occur in May and June (Skjoldal and Rey, 1989; Dalpadado *et al.*, 2020). Thus, in a large data set from the IMR database (>60 000 samples from 8746 stations between 1980 and 2016), the median chlorophyll *a* value for the spring bloom months (May and June) was ~ 1 mg m^−3^, and the 95%-quantile was within 5 mg m^−3^ (Fig. S1 in Supplementary material). Events of considerable clogging due to high phytoplankton biomass are therefore rare, but clogging can nevertheless occur when there are dense spring blooms, especially of the colony-forming algae *Phaeocystis* (Skjoldal and Rey, 1989; Wassmann *et al.*, 1990). Overall, clogging is not expected to have significantly affected our results.

Each sample is split in two halves with a Motoda plankton divider. Dry weight biomass of three size fractions is determined following wet sieving of one half-sample through 2000-, 1000- and 180-μm screens (Hassel *et al.*, 2020; Skjoldal, 2021). The three fractions are denoted large (>2 mm), medium (1–2 mm) and small (<1 mm), where the size limits refer to mesh size of the screens. The second half-sample is preserved with buffered formaldehyde and stored for later species counts.

For copepods and cladocerans, the separation by the screens follows a strict relationship with size of the individuals (Skjoldal, 2021). Thus, the 1-mm (1000 μm) screen starts to collect individuals with width ~0.4 mm and retains nearly all individuals of width ~0.8 mm, with 50% retention at width ~0.6 mm (Skjoldal, 2021, his Fig. 3). This relationship between width and retention was used to estimate the contribution of taxa to each of the three biomass fractions based on average width of the species and stages (see below). The relationship between the width and prosome length of copepods varies with shape but is typically ~1:3 (Pearre Jr., 1980; Skjoldal *et al.*, 2013). Thus, the size range between no and full retention by the 1-mm screen is for a range in prosome length of ~1.2–2.4 mm.

The retention of zooplankton by the 180-μm WP2 net follows a steep logistic function that can be approximated by a linear slope from no retention at ~ 0.1-mm width to full retention at ~ 0.25-mm width (Nichols and Thompson, 1991; Skjoldal *et al.*, 2013, their Fig. 34). Small copepods like *Oithona* and *Microcalanus* have width ~0.2 mm as adults and are sampled to a low degree for the smaller and younger copepodite stages. *Pseudocalanus* spp. are somewhat larger (width ~0.3–0.35 mm) but also for these species, young copepodites are mostly passing through the 180-μm net (Skjoldal *et al.*, 2013). We have reviewed literature on size of copepods and cladocerans and listed mean width and individual weight used for calculating biomass from numbers of individuals in samples (see below and Table S1). In doing so, we have considered the low sampling efficiency for young copepodite stages of small species.

Taxonomic analysis and species counts are made with an adaptive procedure including subsampling, aimed at counting a sufficient number of copepodites of the biomass-dominant *Calanus* species (at least 100 individual copepodites; Hassel *et al.*, 2020). The degree of subsampling for counts of other zooplankton taxa is therefore dictated to large degree by the abundance of *Calanus* species in the samples. Subsampling introduces variance in species counts (Skjoldal *et al.*, 2013), but this effect is difficult to quantify with our data.

The three *Calanus* species (*C. finmarchicus*, *C. glacialis* and *C. hyperboreus*) are counted separately for each of the six copepodite stages. For *Metridia*, *Pseudocalanus* and *Paraeuchaeta* species, counts are made for copepodite stages 1–3 and 4 and 5 combined and for adults (C6, females and males separated). *Metridia longa* and *M. lucens* are counted separately for adults, with *M. longa* as the dominant species in our material. Adults of *Paraeuchaeta* are also identified to species, but *P. norvegica* was the only species recorded in the data set. *Pseudocalanus* occurs with *Pseudocalanus acuspes* and *P. minutus* as the two dominant species in the Barents Sea (Norrbin, 1991). Other copepods (*Acartia, Centropages, Microcalanus, Oithona, Oncaea, Temora*) and cladocerans (*Evadne, Podon*) were counted as sum of copepodite stages, or individuals for cladocerans, and were not identified to species. *Microcalanus* occurs with the species *M. pusillus* and *M. pygmaeus* in the Barents Sea (Norrbin, 1991). *Acartia longiremis* is found in coastal waters of the southern Barents Sea (Norrbin, 1994) and in the Pechora Sea in the southeastern Barents Sea (Dvoretsky and Dvoretsky, 2023). *Centropages* may occur with two temperate-boreal and mostly coastal species, *C. hamatus* and *C. typicus*, which may extend their distributions into the southern Barents Sea (Beaugrand *et al.*, 2007; Dvoretsky and Dvoretsky, 2023). *Temora* is likely *T. longicornis*, which is found in the western and southern Barents Sea (Dvoretsky and Dvoretsky, 2023). The cyclopoid copepod *Oithona* occurs in the Barents Sea with *O. similis* as the most important species, but *O. atlantica* is also found although generally less abundant (Dvoretsky and Dvoretsky, 2009, 2015). *Oncaea* are also cyclopoid copepods, with *Triconia borealis* as the dominant species in the Barents Sea. The dominant species of the cladoceran *Evadne* is *E. nordmanni*.

Individual size (length and width) and weight of the copepod and cladoceran taxa were found through literature review, using the information on likely dominant species described above. The size of *Calanus* species was taken from Aarflot *et al.* (2018) and sources given there. The sources on the size of *Metridia* spp. were Grønvik and Hopkins (1984), Hirche and Mumm (1992) and Halliday (2001), and those for *Paraeuchaeta* were Båmstedt and Matthews (1975) and Bakke (1977). The size of small copepod species was based on Corkett and McLaren (1979), Klein Breteler *et al.* (1982), McLaren *et al.* (1989), Hay *et al.* (1991) and Skjoldal *et al.* (2013). The size of cladocerans was based on Hernroth (1985) and Skjoldal *et al.* (2013). The values of individual weight used to calculate taxa biomass are listed in Table S1.

Biomass for each taxon was separated into the three size fractions based on the results in Skjoldal *et al.* (2021), with proportions allocated in the three fractions listed in Table S1. Small proportions of small taxa (typically ~5% or less) were retained in larger fractions as “contamination” due to incomplete “washing” of samples during the wet sieving process (Skjoldal, 2021). For the biomass-dominant *Calanus* spp., most individuals of copepodite stages C1–C3 were contained in the small fraction (85–100%), most individuals of stages C5 and adults were contained in the medium fraction (80–90%), whereas stage C4 of *C. finmarchicus* was split ~50:50 between the small and medium fractions.

**Fig. 1:**
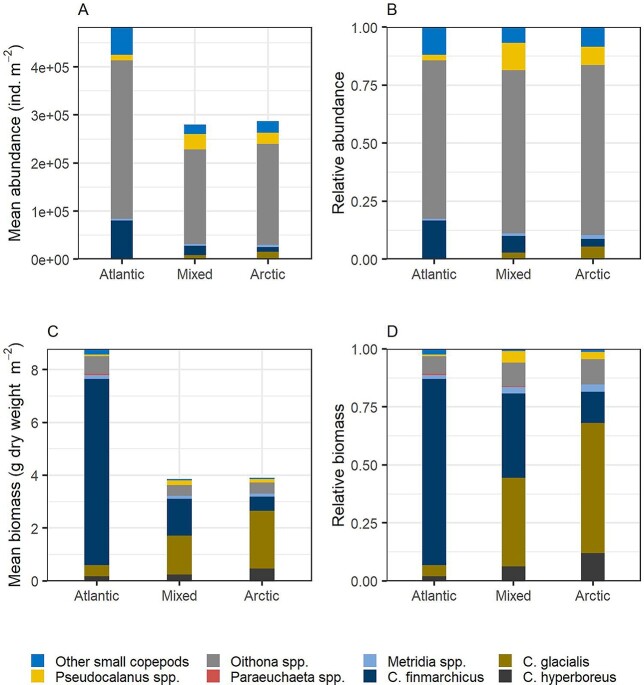
Mean abundance (number of individuals m^−2^) and mean estimated biomass (g dry weight m^−2^) of copepods in Atlantic, mixed and Arctic water masses in the Barents Sea for samples collected in “summer” season (May–September), shown as absolute values (**A** and **C**) and relative values (**B** and **D**).

We have used the same data set of samples as the one used and described by (Aarflot *et al.*, 2018, see their Tables I and II and Fig. 1). We did not include samples obtained with a pump system (“Hufsa”) and MOCNESS, so the number of samples in the present study (*n* = 580) is a little lower than that in Aarflot *et al.* (2018; *n* = 616). The samples were collected over >3 decades (1983–2016) and in different parts of the Barents Sea. We used the division of stations into Atlantic, Arctic and mixed water masses from Aarflot *et al.* (2018), based on temperature at 50-m depth. Most stations were in Atlantic water in the southwestern Barents Sea (445 stations, or 77% of the total), reflecting a high number of samples from regular monitoring at the Fugløya-Bear Island oceanographic transect starting in 1992 and continued annually from 1995 onward (see Skjoldal *et al.*, 2021). A smaller number of stations were from Arctic waters (31 stations, or 5%), while a somewhat larger number (104, or 18%) were from the mixed water masses, including the Polar Front region at the transition between Atlantic and Arctic waters (Ingvaldsen *et al.*, 2021) (Table S2, Fig. S2).

To illustrate the full range of variability, we used all data including the winter period. For a more detailed comparison between the Atlantic, Arctic and mixed water masses, we used data for an extended summer period (May–September), corresponding to the period of significant primary production by phytoplankton (Dalpadado *et al.*, 2020). The “summer” samples made up about half of the total (295 stations) but included larger fractions of the samples from Arctic (26 stations) and mixed (61 stations) water masses (Table S2). Most of the samples were collected in June, August and September.

The data set is heterogenous in time and space since it has resulted from various research and monitoring projects with different objectives. Therefore, we did not examine interannual variability in this study. We note that there is an extensive collection of samples (half-samples for taxonomic analysis) in a repository at IMR which, if processed, can be used to address issues of changes in species composition with time. The central area of the Barents Sea including the polar front region is the core area of distribution of the Barents Sea capelin stock (Skjoldal *et al.*, 2022). The total zooplankton biomass and the ratio between the small and medium size fractions have been shown to fluctuate inversely with the size of the capelin stock (Skjoldal, 2023). We examined the relationship between estimated biomass of small copepods and *Calanus* species in relation to variation in measured zooplankton biomass using linear regression.

Data analyses and figures were done using R (R Core Team, 2016). Linear regressions between calculated biomass of taxa and observed zooplankton biomass were done as ordinary linear regression (OLR). Data on numerical abundance, calculated biomass of taxa and observed zooplankton biomass were log10-transformed. A low value of 10^−6^ was added to the numerical abundance and biomass data prior to log-transformation. A log10 value of −6 is therefore equivalent to zero individuals m^−2^.

## RESULTS

### Abundance

*C. finmarchicus* was recorded in all samples (580), while *Oithona* spp. were recorded in nearly all (97%; Table I). *Metridia, Microcalanus* and *Pseudocalanus* spp. were recorded in 82–95% of the samples, while *C. glacialis* and *C. hyperboreus* were recorded in 57% and 52% of the samples, respectively. The remaining taxa were recorded in from 3% (*Podon* spp.) to 32% (*Acartia* and *Paraeuchaeta* spp.) of the samples (Table I).

**Table I:** Frequency of occurrence (% of the total number of samples) and mean, median and maximum abundance and biomass per m^2^ surface area, for copepod and cladoceran taxa across the sample series (n = 580) from the Barents Sea

Species	Frequency (%)	Abundance (no. of individuals m^−2^)			Biomass (g dry weight m^−2^)		
		Mean	Median	Maximum	Mean	Median	Maximum
*C. finmarchicus*	100	37 279	10 624	988 160	3.596	1.29	46.25
*C. glacialis*	57	2315	24	123 392	0.472	0.01	13.68
*C. hyperboreus*	52	219	8	6672	0.154	0.00	5.57
*Metridia* spp.	88	2954	928	57 984	0.140	0.06	2.21
*Paraeuchaeta* spp.	32	64	0	8528	0.031	0.00	1.70
*Pseudocalanus* spp.	95	12 438	2528	423 936	0.079	0.02	1.93
*Microcalanus* spp.	82	27 899	8832	879 040	0.042	0.01	1.32
*Acartia* spp.	32	2942	0	370 176	0.029	0.00	3.70
*Centropages* spp.	7	316	0	27 680	0.006	0.00	0.55
*Temora* spp.	12	1509	0	270 336	0.030	0.00	5.41
*Oithona* spp.	97	223 536	68 096	14 766 080	0.447	0.14	29.53
*Oncaea* spp.	45	3181	0	150 528	0.013	0.00	0.60
*Evadne* spp.	6	804	0	271 872	0.006	0.00	1.90
*Podon* spp.	3	112	0	24 576	0.001	0.00	0.17
Sum		315 567			5.05		

*Oithona* spp. was the most abundant taxon with a mean of 224 000 individuals (copepodites) m^−2^ across the full set of samples (Table I). The second most abundant taxon was *C. finmarchicus* (37 000 ind. m^−2^), followed by *Microcalanus* spp. (28 000 ind. m^−2^). *Pseudocalanus* spp. were recorded with mean abundance of 12 000 ind. m^−2^ and *Metridia* spp. with 3000 ind. m^−2^ (Table I). The cladocerans were recorded with low mean abundances of 800 and 100 ind. m^−2^ for *Evadne* and *Podon*, respectively (Table I).

The distribution of abundance among samples was highly skewed on a linear scale, with median abundances being a small fraction (0.2–0.3) of mean abundances for the taxa with high frequency of occurrence (Table I). The distributions were balanced and symmetrical after log-transformation for the common taxa (*C. finmarchicus, Metridia, Pseudocalanus, Microcalanus, Oithona*), resembling a log-normal distribution (Fig. S3A). For species with a frequency of occurrence <50%, the median abundance was equal to zero, while for the least frequently occurring taxa (*Centropages, Temora, Evadne, Podon*; present in 3–12% of all samples), the box-whisker diagram showed only individual data points as “outliers,” with boxes hidden in the zero value (10^−6^; Fig. S3A).

When broken down by water masses and using samples for an extended summer season (May–September), abundance showed a remarkable similarity between the Atlantic, mixed (“polar front”) and Arctic water masses (Fig. 1A and B, Table S3). While the mean total abundance of copepods was higher in Atlantic water (480 000 ind. m^−2^) compared to mixed and Arctic waters (~280 000 ind. m^−2^), *Oithona* spp. dominated with ~70% of total abundance in each of the three water masses (68–73%). The *Calanus* species taken together varied from 17% of abundance in Atlantic water to 9% in Arctic water. This change reflected a high mean abundance of *C. finmarchicus* (78 000 ind. m^−2^) in Atlantic water, whereas *C. glacialis* dominated in Arctic water (mean abundance 15 000 ind. m^−2^; Fig. 1A). *Pseudocalanus* spp. were relatively more abundant in the mixed and Arctic waters (12% and 8%) compared to Atlantic water (2%). The group of “other small copepods” (*Acartia, Centropages, Microcalanus, Oncaea* and *Temora*) showed an opposite pattern with 12% of total abundance in Atlantic water and 7–8% in mixed and Arctic waters (Fig. 1B).

Highest mean abundances of the two cladoceran taxa (2000 and 300 ind. m^−2^) were found in Atlantic water, and no cladocerans were recorded in Arctic water (Table S3).

### Biomass

**Fig. 2:**
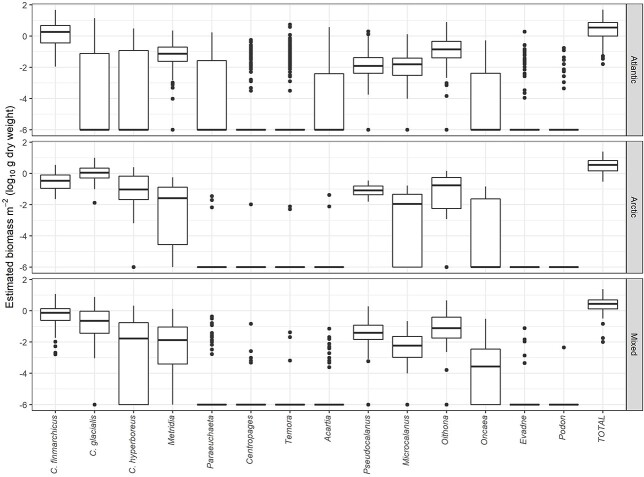
Box-whisker diagrams of calculated biomass of 12 copepod and 2 cladoceran taxa for stations located in Atlantic (upper panel), Arctic (middle panel) and mixed water masses (lower panel). Data are log10-transformed, with a low value of 10^−6^ added to the numbers; the log10 value of −6 is therefore equivalent to 0 (zero). The horizontal bars, boxes, whiskers and individual points are median values, 25–75 percentiles, 5–95 percentiles and “outliers,” respectively.

The distribution of biomass among samples for each taxon was similar to that for abundance, since biomass is calculated from the abundance data applying a scaling factor (mean individual weight), or a set of scaling factors for copepod taxa where different copepodite stages are enumerated (Fig. 2 and S3). However, the relative role of taxa shifted from abundance to biomass, reflecting the different size of copepods and cladocerans (Fig. 1). The highest estimated mean biomass for the summer data was for *C. finmarchicus* in Atlantic water (7.0 g dw m^−2^), followed by *C. glacialis* in Arctic water (2.2 g dw m^−2^) (Fig. 1C, Table S3). The biomass of *C. hyperboreus* was relatively low (0.2 g dw m^−2^ in Atlantic and mixed water and 0.5 g dw m^−2^ in Arctic water). The third most important taxon in terms of biomass was *Oithona* spp. with mean estimated biomass of 0.4–0.7 g dw m^−2^ (highest in Atlantic water; Table S3). The mean estimated biomass values of *Metridia* spp. was 0.11–0.16 g dw m^−2^ in the three water masses, while *Pseudocalanus* and *Microcalanus* had mean biomass values of 0.07–0.19 and 0.02–0.06 g dw m^−2^, respectively (Table S3). Other copepod taxa had low biomass (0.06 g dw m^−2^ or less), as had the two cladocerans (0.01 and 0.002 g dw m^−2^ for *Evadne* and *Podon*, respectively, in Atlantic water) (Table I and S3).

The relative biomass distribution, like abundance, was remarkably similar across the three water masses (Fig. 1D and S4, Table S3). *Calanus* species made up 81–87% of the estimated total biomass of copepods in all water masses. The contribution by *C. finmarchicus* decreased from 80% in Atlantic water to 14% in Arctic water, while *C. glacialis* increased from 5% to 56% between the same water masses (Fig. 1D). The relative contribution by *C. hyperboreus* also increased, from 2 to 12%. The numerically dominant *Oithona* species made up 8–11% of the estimated biomass of copepods. *Metridia* spp. made up 2–3%, *Pseudocalanus* spp. 1–5% (lowest in Atlantic water), *Paraeuchaeta* spp. 0.04–0.5% (lowest in Arctic water) and the group of other small copepods 1–2%.

### Contribution of copepods and cladocerans to biomass of the three size fractions

**Fig. 3:**
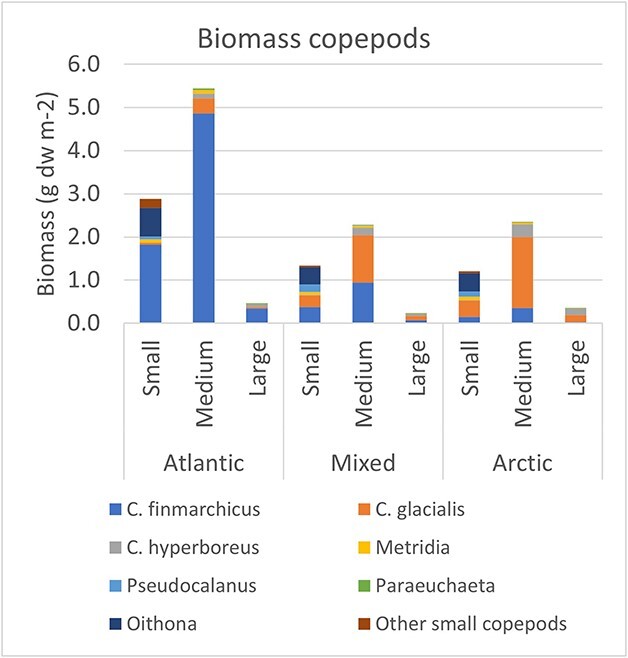
Contribution of calculated biomass by copepods to the three size fractions used in routine measurements of biomass in the Barents Sea (small—< 1 mm; medium—1–2 mm; large—> 2 mm). Results are shown for the extended summer data (May–September) for Atlantic, mixed and Arctic water masses. The estimated biomass values of taxa were split into the three size fractions by proportional coefficients given in Table S1, based on results in Skjoldal *et al.* (2021).

*Calanus* species contributed nearly all (~98%) of the calculated biomass of copepods of the medium size fraction, along with a small contribution by *Metridia* (Fig. 3). *Calanus* also contributed most of the biomass of the large fraction (95–98%), along with *Metridia* (~2%) and *Paraeuchaeta* (0.4–2.8%). There was a pronounced shift from dominance of *C. finmarchicus* in Atlantic water to dominance by *C. glacialis* in Arctic water, which was particularly evident for the medium and large size fractions (Fig. 3). The small copepod taxa with prosome length of ~1 mm or less, including *Oithona*, *Pseudocalanus* and *Microcalanus,* were assigned almost exclusively to the small fraction where their biomass combined (0.6–0.9 g dw m^−2^) contributed 32–49% of the calculated biomass for the three water masses. The absolute biomass of *Oithona* was slightly higher in the small size fraction in Atlantic compared to Arctic water masses, but their relative contribution was higher in Arctic water due to lower contribution from *Calanus* to this size fraction. Overall, *Calanus* made up a large proportion of the biomass (45–65%) of the small size fraction from the young copepodite stages, with a small contribution (2–6%) by *Metridia* (Fig. 3). Cladocerans were also assumed to be mainly in the small fraction, but their contribution was low (0.6% in Atlantic water and none in Arctic water).

The relative distribution of estimated biomass of copepods in the three size fractions was remarkably similar between the water masses (Fig. 3). The small, medium and large fractions comprised 33, 62 and 5% of the biomass, respectively, in Atlantic water, while for mixed water, the proportions were 35, 59 and 6%, and for Arctic water, 31, 60 and 9%.

**Fig. 4:**
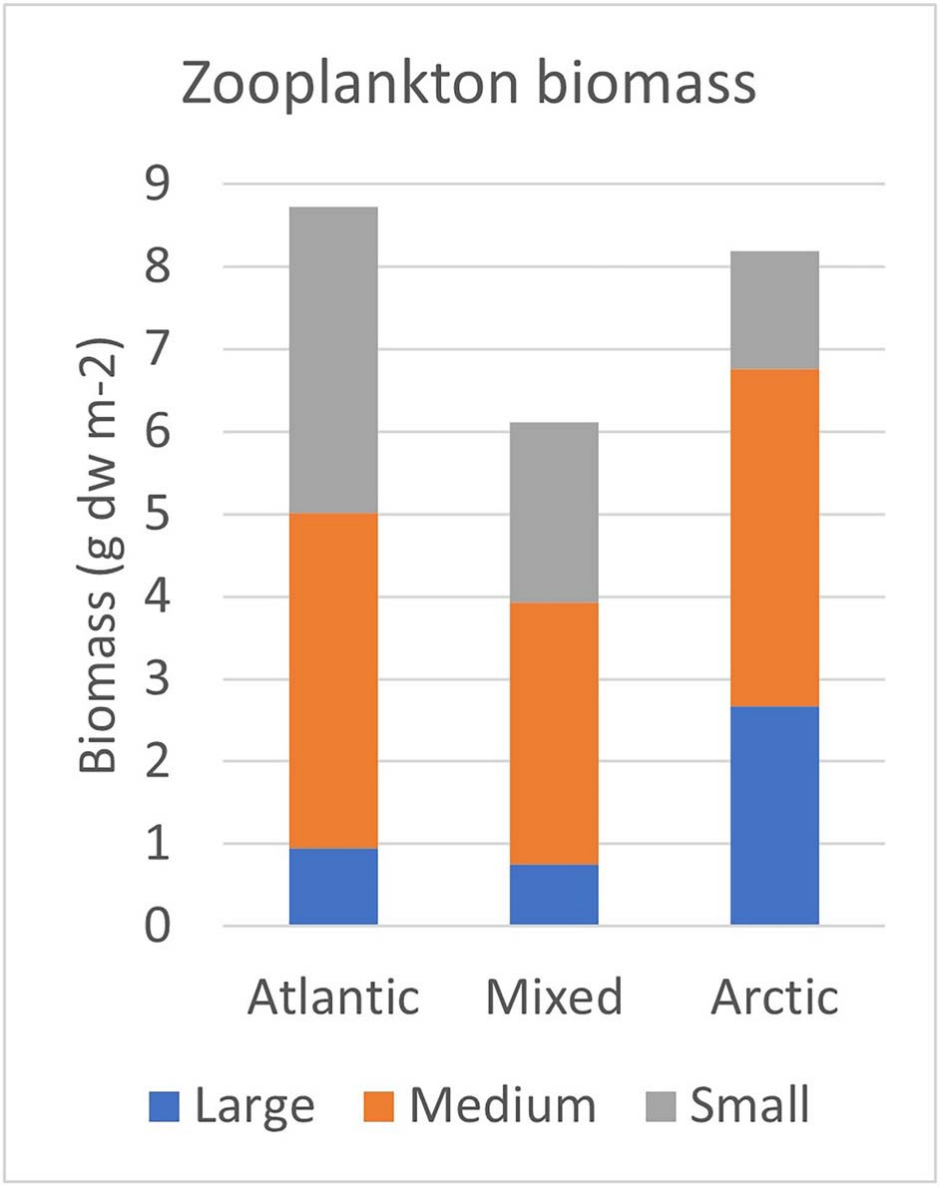
Measured biomass as mean dry weight (dw) in three size fractions for the half-samples complementary to the extended summer (May–September) data set with species counts and estimated biomass of copepods and cladocerans (see Fig. 3).

The measured biomass of the set of half-samples that were complementary to the half-samples used for species counts (Fig. S5) varied from a mean of 6.1 g dw m^−2^ for mixed water to a mean of 8.7 g dw m^−2^ for Atlantic water for the extended summer data (Fig. 4, Table S4). About half of the measured biomass was contained in the medium size fraction in each of the water masses (47–52%), whereas there was an increase from 11% to 33% between the Atlantic and Arctic waters for the large fraction and a corresponding decrease from 43% to 18% for the small fraction (Fig. 4).

**Fig. 5:**
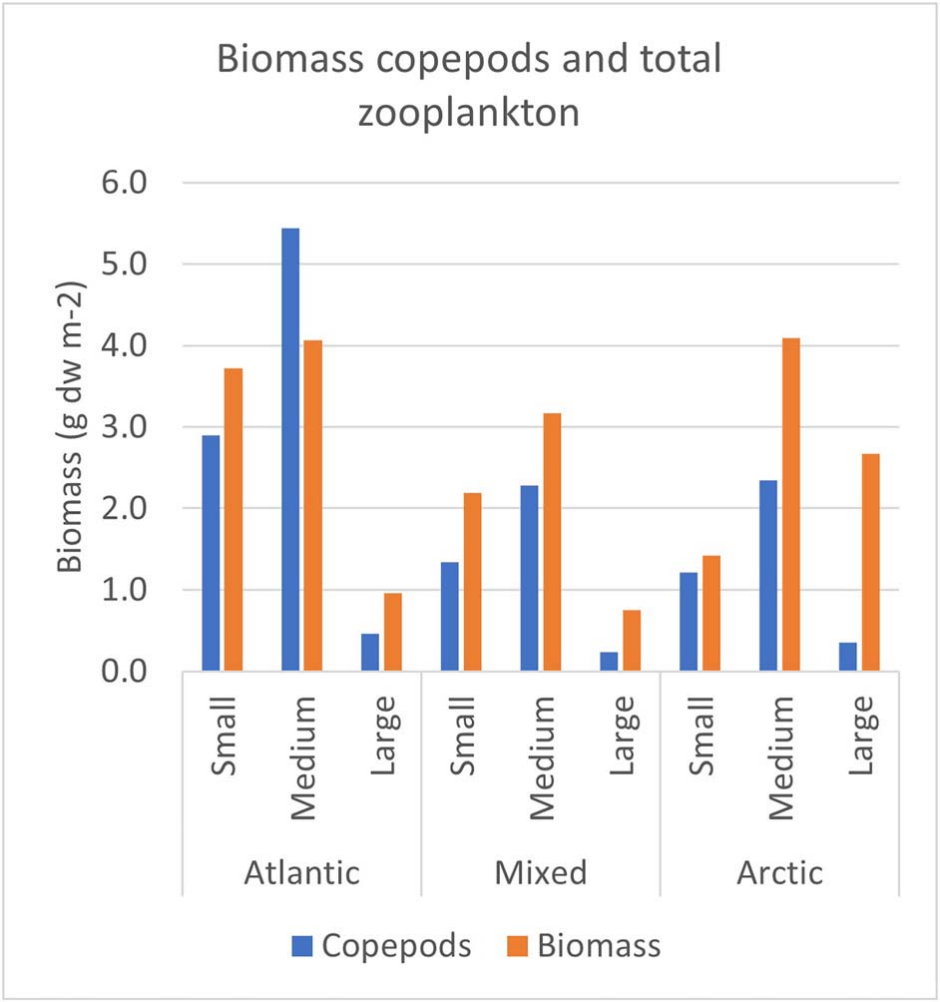
Estimated biomass of copepods compared to measured zooplankton biomass of three size fractions, based on mean values for the extended summer (May–September) data sets for Atlantic, mixed and Arctic waters (see Figs 3 and 4).

Comparing the estimated biomass of copepods with the observed biomass for the three size fractions (Fig. 5) reveal that the estimated biomass of copepods was about equal to the measured total biomass in Atlantic water but lower by a third to a half (37% and 52%) of the total biomass measured in mixed and Arctic waters. The calculated biomass of copepods for the medium fraction exceeded the measured biomass (by 34%) in Atlantic water, while it was lower (by 28% and 43%) in mixed and Arctic waters (Fig. 5). The estimated copepod biomass was lower than measured biomass for the small and large fractions, making up 61–85% and 13–48%, respectively.

### Small copepods versus *Calanus*

The ratio of mean biomass of small copepods (sum of *Oithona*, *Pseudocalanus* and other small copepods) to mean biomass of the *Calanus* species varied from 0.12 (Atlantic) to 0.2 (mixed water; 0.19 in Arctic water) for the summer data (Fig. 3, Table S3). The corresponding ratios of small copepods to large calanoid copepods (*Calanus* plus *Metridia* and *Paraeuchaeta*) were 0.12, 0.19 and 0.18 for Atlantic, mixed and Arctic waters, respectively.

**Fig. 6:**
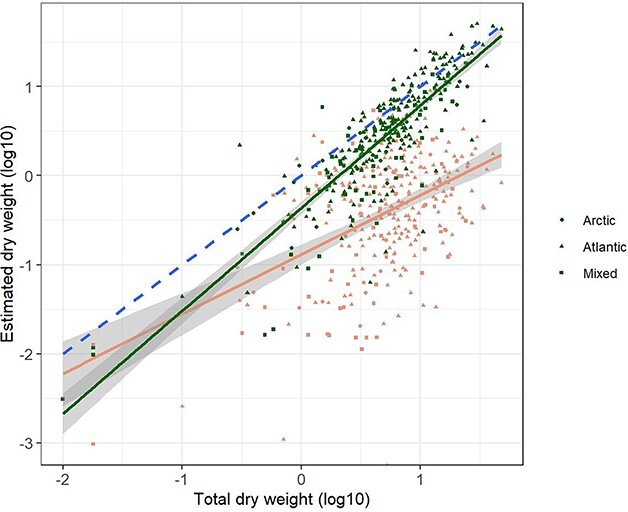
Scatter plots and regression lines with 95% confidence bands for estimated biomass of *Calanus* (green) and of the sum of small copepod species (red) versus the measured total biomass for the extended summer data (May–September). Individual values are indicated with different symbols for water masses. The values are log10-transformed. Equations for the regression lines are given in Table II. The broken blue line shows the 1:1 ratio.

Linear regressions showed that the estimated biomass of both *Calanus* and the sum of small copepod species [*Acartia*, *Centropages*, *Microcalanus, Pseudocalanus*, *Temora, Oithona* and *Oncaea* (*Triconia*)] were positively related to the measured total zooplankton biomass (*P* < 0.001) (Fig. 6, Table II). Total measured biomass explained a higher degree of the variation in estimated biomass of *Calanus* (*R*^2^ = 0.73) than the variation in estimated biomass of small copepods (*R*^2^ = 0.35). Including water mass as a categorical grouping variable with two levels (Atlantic and mixed plus Arctic) increased the explanatory power of the regression for *Calanus* versus total measured biomass (*R*^2^ = 0.79), with a significant difference in intercept (higher for Atlantic) but not for slope (Table II). Water mass had no significant effect for the regression of small copepods.

**Table II:** Results from ordinary linear regressions of estimated biomass of Calanus spp. and small copepods as a function of the total observed zooplankton biomass for data collected during an extended summer period (May–September)

log_10_(*Calanus* biomass) = *a* + *b*(log_10_(total biomass))					
		Estimate	Std. error	*P*-value	*R* ^2^
	*a* (intercept)	−0.365	0.036	<0.001	
	*b* (slope)	1.153	0.042	<0.001	
					0.72
log_10_(*Calanus* biomass)_W_ = *a*_W_ + *b*_W_(log_10_(total biomass))					
Water mass		Estimate	Std. error	*P*-value	*R* ^2^
Arctic & mixed	*a*	−0.370	0.036	<0.001	
	*b*	1.026	0.052	<0.001	
Atlantic	*a*	−0.269	0.041	0.01	
	*b*	1.087	0.058	0.30	
					0.79
log_10_(small copepods) = *a* + *b*(log_10_(total biomass))					
		Estimate	Std. error	*P*-value	*R* ^2^
	*a* (intercept)	−0.983	0.029	<0.001	
	*b* (slope)	0.686	0.039	<0.001	
					0.34
log_10_(small copepods)_W_ = *a*_W_ + *b*_W_(log_10_(total biomass))					
Water mass		Estimate	Std. error	*P*-value	*R* ^2^
Arctic & mixed	*a*	−1.011	0.062	<0.001	
	*b*	0.687	0.089	<0.001	
Atlantic	*a*	−0.978	0.070	0.64	
	*b*	0.688	0.100	0.99	
					0.34
log_10_(small copepods/*Calanus* spp.) = *a* + *b*(log_10_(total biomass))					
		Estimate	Std. error	*P*-value	*R* ^2^
	*a* (intercept)	−0.572	0.074	<0.001	
	*b* (slope)	−0.434	0.086	<0.001	
					0.08

Two versions of each model are presented; one with all data irrespective of water mass and one where water mass (*W*) was included as a categorical grouping variable with two levels: Arctic & mixed and Atlantic. The second set of *P*-values for the models with water masses included gives the probability for significant differences in intercept and slopes for the two cases (Atlantic versus Arctic & mixed). Also included is the regression equation for the log10 ratio of biomass of small copepods to biomass of *Calanus* versus total observed zooplankton biomass.

**Fig. 7:**
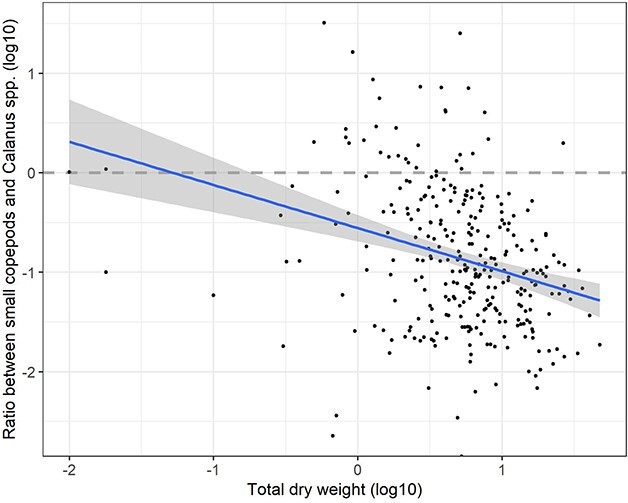
Scatter plot and regression line with 95% confidence band for the biomass ratio of small copepod species to *Calanus* spp. versus measured total zooplankton biomass (both are log10-transformed). Regression equation is given in Table II.

**Fig. 8:**
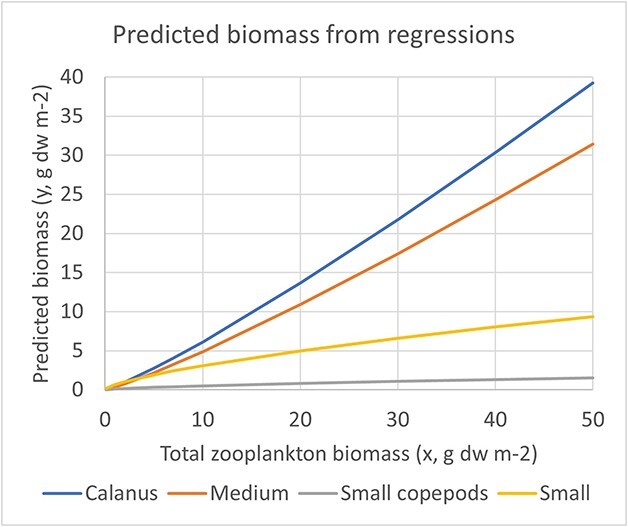
Regression lines for estimated biomass of *Calanus* species and sum of small copepods [*Oithona, Pseudocalanus, Microcalanus, Acartia, Centropages, Oncaea (Troconia)* and *Temora*] versus measured total zooplankton biomass from this study (equations in Table II) and regression lines for measured biomass of the medium and small size fractions versus measured total zooplankton biomass from the extended data set from autumn monitoring in the Barents Sea reported by Skjoldal *et al.* (2022). Regression equations (*y* = *a* + *bx*) for log10-transformed data are *a* = −0.468 and *b* = 1.157, *R*^2^ = 0.83 for the medium fraction; *a* = −0.196, *b* = 0.687, *R*^2^ = 0.49 for the small fraction (Skjoldal and Sperfeld, unpublished results).

The slope was steeper for *Calanus* than for small copepods (Fig. 6; 1.15 versus 0.69), reflecting an increase in the biomass ratio of small copepods to *Calanus* in the direction of decrease in total biomass (Fig. 7). The regression slope for the log10 ratio (small copepods/*Calanus*) versus log10 total biomass was −0.43 (Table II). The regression slopes for *Calanus* and small copepods were nearly identical to slopes obtained for biomass of the medium and small size fractions versus total biomass for a large data set from autumn monitoring surveys in the Barents Sea (Skjoldal *et al.*, 2022). The regression line for *Calanus* was higher than that for the medium biomass fraction by 25% (Fig. 8). The regression line for small copepods was placed lower than that of the small biomass fraction, with predicted biomass being 16% of the biomass of the small fraction (Fig. 8).

## DISCUSSION

### Biomass dominance of *Calanus*

Aarflot *et al.* (2018) reported a strong dominance of the *Calanus* species for the zooplankton biomass of the Barents Sea, with a shift in dominance from *C. finmarchicus* in the Atlantic water to *C. glacialis* in Arctic water. We have here extended the study of Aarflot *et al.* (2018) by estimating biomass of other copepods, allowing a broader comparison of biomass of *Calanus* in relation to biomass of small copepods and other species of the copepod community, as well as to measured biomass in size fractions. We confirm the strong dominance of the *Calanus* species for the zooplankton biomass of the Barents Sea. Small copepod species, dominated by *Oithona* spp., make up only ~15–20% of the estimated copepod biomass, with *Calanus* species making up most of the other ~80–85% (Fig. 3). Before we discuss the new biomass results for small copepods, we provide a reevaluation of the estimated biomass of *Calanus*.

The calculated biomass of *Calanus* in the medium size fraction for the Atlantic water mass is larger (by 34%) than the recorded biomass (Fig. 5), which suggests that the individual weights used to convert from numbers to biomass are on the high side. We used a weight of 250 μg (dry weight) for copepodite stage C5 of *C. finmarchicus*, which makes up most of the calculated biomass of the species (66%). This value for C5 was based on an average of mean values from nine published sources (244 μg, SD 144; see Fig. 1A of Aarflot *et al.*, 2018), rounded up to 250 μg, which was the same as a mean value found by Tande (1982) for C5 *C. finmarchicus* in a fjord in northern Norway adjacent to the southern Barents Sea. By comparison, Blachowiak-Samolyk *et al.* (2008) used a weight of 274 μg for C5 *C. finmarchicus* to calculate biomass in samples from Svalbard. The size of *Calanus* copepodites can vary much, with individual C5 *C. finmarchicus* varying roughly by a factor 2 in length and up to an order of magnitude in weight (for examples, see Marshall *et al.*, 1934 and Fig. S2 in Aarflot *et al.*, 2018). The mean value (244 μg) of the nine sources referred to above was strongly influenced by an exceptionally high value from the Fram Strait (630 μg), being more than twice the second highest value. Excluding this high value, the mean for the remaining eight sources was 201 μg (SD 50).

There is a general inverse relationship between size of *Calanus* and ambient temperature, with the second generation (G2) in summer being smaller than the first spring generation (G1) by 5–10% in length (Marshall *et al.*, 1934; Wiborg, 1954; McLaren *et al.*, 2001). Thus, Wiborg (1954) found a decrease in prosome length of C5 *C. finmarchicus* from 2.57 mm (SD 0.13) in spring/early summer to 2.30 mm (SD 0.12) in autumn at a coastal station (Eggum) just south of the entrance to the Barents Sea. Very similar results were found by McLaren *et al.* (2001) for *C. finmarchicus* on the Scotian shelf, with decrease in length from 2.55 mm in April–May to 2.3 mm in June to November. Using a weight–length regression from Hirche and Mumm (1992), the results of Wiborg (1954) give a decrease in weight from ~230 to 160 μg, or a decrease by ~30% (for a decrease in length by ~10%). A reduction of the applied weight of *C. finmarchicus* by 30% (from 250 to 175 μg for C5 copepodites and similar reductions by 30% for the other stages) would lower the calculated biomass for this species in the Atlantic water mass from 7.0 to 4.9 g dw m^−2^ and its contribution to calculated biomass of all copepods from 80% to 74%.

There has been a pattern of increased or maintained high biomass of zooplankton in the inflow region of Atlantic water in the southwestern Barents Sea (Aarflot *et al.*, 2018; Skjoldal *et al.*, 2022). This has corresponded to high abundance of *C. finmarchicus*, which has been interpreted to reflect increased occurrence of a second (G2) generation in summer due to a warming trend in the recent decades (Skjoldal *et al.*, 2021, 2022; see also Strand *et al.*, 2020). Applying a lower weight by 30% for *C. finmarchicus* brings the calculated biomass of copepods to the same level as the recorded biomass of the medium fraction (4.0 versus 4.1 g dw m^−2^). While *Calanus* is clearly dominating, the medium fraction also contains meroplanktonic larvae (e.g. polychaetes and echinoderms) and small individuals of chaetognaths (Skjoldal, 2021). Thus, the adjusted value of 30% reduction for *C. finmarchicus* may still be somewhat on the high side for the recent warmer conditions with possibly smaller individuals of *C. finmarchicus*.

Aarflot *et al.* (2018) found a strict relationship between calculated biomass of *Calanus* versus measured total zooplankton biomass (*R*^2^ = 0.79; see Fig. 6 and Table II). A similar strict relationship between biomass of the medium size fraction versus total biomass was found for an extended data set including all monitoring stations (*n* = 4 543) collected in the period 1989–2020 (*R*^2^ = 0.83; Skjoldal and Sperfeld, unpublished results). Both regressions (ordinary linear regression) had slopes >1 (1.153 and 1.157), indicating increased relative importance of *Calanus* and the medium fraction with increasing total biomass. In both cases, data were log-transformed. Comparing the two regressions reveals that the relationship for *Calanus* is higher than that for the medium fraction by 25% (Fig. 8). We estimate that ~70% of the biomass of *Calanus* sits in the medium size fraction, which brings the two regressions in close agreement but does not allow for biomass of other groups such as meroplankton and chaetognaths. This again suggests that the estimated biomass of *Calanus* is on the high side.

The mean biomass values for *Calanus glacialis* and *C. hyperboreus* were low in the Atlantic water where these species are relatively scarce, but increased to mean values of 2.2 and 0.5 g dw m^−2^ in Arctic water for summer data of the two species (Table S3). We note that the mean biomass of *C. glacialis* in Arctic waters is similar too or on the low side compared to other studies (see Aarflot *et al.*, 2018, their Table S3). Thus, Blachowiak-Samolyk *et al.* (2008) found a mean value of 4.9 g dw m^−2^ in Arctic waters near Svalbard. The estimated biomass of copepods, dominated by *C. glacialis* (Fig. 3), made up 57% compared to the measured biomass in the medium fraction in our data for Arctic water (Fig. 5). This allows for presence of plankton forms other than copepods, but it is difficult to evaluate if our estimate of biomass for *C. glacialis* is on the low side. However, we note that we have used a lower mean size of *C. glacialis* to estimate biomass than that used by Blachowiak-Samolyk *et al.* (2008) (600 versus 780 μg dw for the biomass-dominant stage C5). The biomass of *C. hyperboreus* is generally low in the Barents Sea, which probably reflects its vulnerability to predation from visual predators such as capelin (Aarflot *et al.*, 2022; Langbehn *et al.*, 2023). See Aarflot *et al.* (2018) for more information and comparison of biomass of these two *Calanus* species in different regions.

### Biomass of other large calanoid copepods

The *Calanus* species are by far the dominant large calanoid copepods in the Barents Sea. Two other taxa fall into this category of copepods—*Metridia* and *Paraeuchaeta*. *Metridia* spp. had mean abundance and biomass of ~4000 ind. m^−2^ and ~0.15 g dw m^−2^ and made up 2–3% of the calculated weight of copepods (Fig. 3). Previous studies in the Barents Sea have found the abundance of *Metridia* to be an order of magnitude lower than the two dominant *Calanus* species (Hassel, 1986; Skjoldal *et al.*, 1987; Falk-Petersen *et al.*, 1999). *M. longa* is common and widespread in northern boreal and Arctic waters, including the central Arctic Ocean where its biomass is typically 0.5–1 g dw m^−2^ in Nansen Basin north of the Barents Sea (Kosobokova and Hirche, 2009; Skjoldal, 2022). It has a more omnivorous diet compared to the *Calanus* species, with a more prolonged reproduction period within an annual life cycle (Grønvik and Hopkins, 1984).

*Paraeuchaeta* spp. are large carnivorous copepods. We found them with low abundance and biomass (mean 0.03 g dw m^−2^) in our study, making up <1% of the biomass of copepods. A reason for their low importance in the Barents Sea could be vulnerability to predation by visual fish predators due to their large size. This makes them easy targets for visual predators in shallow shelf waters (Langbehn *et al.*, 2023) and could explain their tendency to be mesopelagic (Mauchline, 1995).

### Biomass of small copepods

The small copepods belong to two different types: calanoids and cyclopoids. The cyclopoid *Oithona* spp. were most abundant, with a mean biomass of ~0.5 g dw m^−2^, or ~10% of the calculated biomass of copepods (Figs. 1 and 2). The dominant species in the Barents Sea is *Oithona similis* (Dvoretsky and Dvoretsky, 2015), which has been considered the most numerous copepod species globally (Gallienne and Robins, 2001). The prosome length of *O. similis* is ~0.5 mm for stage C5 and adult females (Sabatini and Kiørboe, 1994; Castellani *et al.*, 2007), and they are contained in the small size fraction (Skjoldal, 2021). Our biomass values for *Oithona* are comparable to or on the high side of values found in other northern waters (Dvoretsky and Dvoretsky, 2015, see compilation in their Table 5). Thus, Blachowiak-Samolyk *et al.* (2008) found a mean biomass value of 0.31 g dw m^−2^ (SD 0.20) for stations in northern Svalbard waters.

The taxon *Oncaea* is dominated by the species *Triconia borealis*, which was previously known as *Oncaea borealis* (Böttger-Schnack, 1999). We found low biomass of *Oncaea* in our material (~0.02 g dw m^−2^). Blachowiak-Samolyk *et al.* (2008) found similarly low values (mean 0.02 g dw m^−2^) in northern Svalbard waters, while Dvoretsky and Dvoretsky (2023) found even lower values (<0.001 g dw m^−2^) in the shallow Pechora Sea. Thus, it seems that *T. borealis* and other *Oncaea* species play a small role in the Barents Sea ecosystem.

Among the small calanoid species, *Pseudocalanus* and *Microcalanus* were the most important, although they occurred with low biomass (mean values of ~ 0.1 and 0.05 g dw m^−2^, respectively). For *Pseudocalanus*, this is similar to the mean value (0.09 g dw m^−2^) found by Blachowiak-Samolyk *et al.* (2008) for northern Svalbard waters, while our value for *Microcalanus* is somewhat lower than their value (0.13 g dw m^−2^). Prosome length of the two dominant *Pseudocalanus* species is ~1 mm for the adult stage, with *P. minutus* being slightly larger than *P. acuspes* (Frost, 1989; Norrbin, 1991). The body width is ~0.4 mm for a length of 1 mm, and most individuals pass the 1-mm screen and are found in the small fraction (Skjoldal, 2021). *Microcalanus pusillus* and *M. pygmaeus* are smaller species with prosome length of 0.5–0.6 mm. They tend to be distributed in deeper water (Norrbin, 1991), which may be a reason why *Microcalanus* are scarce in the Barents Sea.

The remaining small calanoid taxa, *Acartia*, *Centropages* and *Temora*, were recorded infrequently (7–32% of samples) with low abundances and very low estimated biomass (0.06 g dw m^−2^ or less). *Acartia clausi, A. longiremis, Centropages hamatus, C. typicus* and *Temora longicornis* are coastal and more southern (temperate and boreal) species that, e.g. are common in the North Sea (Hay *et al.*, 1991). The three taxa were found mainly in Atlantic water with few records from stations in Arctic water (Fig. 2). This suggests that these small copepods occur as extensions from more southern distributions into the southern Barents Sea, where they are found with neritic distribution, e.g. in the shallow Pechora Sea (Dvoretsky and Dvoretsky, 2023; mean biomass 0.01–0.04 g dw m^−2^).

### Small copepods are undersampled and their biomass is underestimated

Small copepods with prosome length of ~0.5–1 mm as adults, have body width of ~0.2–0.4 mm. Median (50%) retention with a 180-μm meshed net is at width equal to the mesh size, or ~0.2 mm (Skjoldal *et al.*, 2013). Small forms such as *Oithona, Oncaea* and *Microcalanus* are therefore only partially sampled by the 180-μm net, while larger forms such as *Pseudocalanus, Acartia* and *Temora* are nearly quantitatively sampled for the adult stages (for width >0.3 mm; Skjoldal *et al.*, 2013). The youngest copepodite stage (C1) is smaller than adults by a factor ~0.2–0.3, giving them width of ~0.05–0.1 mm, suggesting that all of them would pass through the 180-μm net (Skjoldal *et al.*, 2013). Stage C3 is roughly half the length of adults, and their width would be ~0.1–0.2 mm; C3 of small species (e.g. *Oithona*) would still be too small to be sampled, while C3 of the larger species among the small copepods (e.g. *Pseudocalanus*) would be sampled at roughly 50% retention. This serves to illustrate the undersampling of small copepods dependent on individual size across copepodite stages and species. Our calculated biomass of 0.6–0.9 g dw m^−2^ for the small copepod species, or ~15% of the biomass of all copepods (Fig. 1D), is therefore an underestimate due to this bias.

Pasternak *et al.* (2008) denoted zooplankton in the size range 0.2–0.5 mm in length as sub-mesozooplankton, noting that they are technically in the size range defined as mesozooplankton (0.2–20 mm; Lenz, 2000), although being too small to be collected quantitatively with traditional plankton nets with 180- or 200-μm mesh. This group included copepod nauplii and small copepodites. Comparing samples of sub-mesozooplankton obtained with water bottles (30-L Niskin) with 180-μm net samples gave higher abundance by factor 4–56, with biomass values of ~ 0.3–2.5 g dw m^−2^ (converted from carbon by factor 2) for 10 out of 12 stations in the Barents Sea (Pasternak *et al.*, 2008; two more stations had much higher values of ~ 8–15 g dw m^−2^ driven by high abundance of appendicularians). Reigstad *et al.* (2011) presented values of sub-mesozooplankton biomass of ~0.3–2 g dw m^−2^ (converted from carbon) for six stations in the northern Barents Sea.

### Contribution by small copepods to biomass of the small size fraction

The mean biomass as sum of the small copepod species (excluding young and small copepodite stages of *Calanus* spp.) was 0.6–0.9 g dw m^−2^ for the three water masses, comparable to the mean value (0.57 g dw m^−2^) found by Blachowiak-Samolyk *et al.* (2008) for northern Svalbard waters. *Oithona* spp. were dominant in both studies (0.4–0.7 g dw m^−2^ in the present study vs. 0.32 g dw m^−2^) followed by *Pseudocalanus* spp. (0.07–0.19 vs. 0.09 g dw m^−2^). Overall, our biomass profile across the copepod species (Fig. 1C and D, Fig. S4) was quite similar to the results of Blachowiak-Samolyk *et al.* (2008; Fig. S6), with the exception of higher biomass of *Calanus* spp. in the Svalbard study (88% of estimated copepod biomass vs. 81–87% in our study) driven by higher biomass of *C. glacialis* and *C. hyperboreus* (4.9 and 1.1 g dw m^−2^, respectively).

The biomass of the small copepod species was positively correlated with the variation in total zooplankton biomass (Fig. 6). The slope of the regression of biomass of small copepod species vs. measured total biomass was <1 and nearly identical to the slope of a regression of biomass of the small size fraction vs. total biomass (0.686 and 0.687; Fig. 8, Table II). This suggests a strict proportionality between estimated biomass of the small copepod species and biomass of the small fraction.

The *Calanus* species are considered large calanoids, but they are small copepods when they are nauplii and young copepodites. Thus, copepodite stage C1 of *C. finmarchicus* is of similar size as adult *Oithona* or *Microcalanus*, while stage C3 is comparable in size to adult *Pseudocalanus*. Stages C1–C3 of *C. finmarchicus* and *C. glacialis* have width < 0.4 mm and are contained in the small size fraction (Skjoldal, 2021) along with the small copepod species. We calculated that *Calanus* made up ~half or more (45–65%, 0.5–1.9 g dw m^−2^) of the biomass of the small fraction (Fig. 3). The largest contribution in Atlantic water was by stage C4 of *C. finmarchicus*, which we considered split ~50:50 between the small and medium fractions based on Skjoldal (2021). There is uncertainty associated with applying a fixed mean value since the separation is sensitive to small changes in size of the C4 copepodites, which were found to vary from ~30% to 70% in the small fraction across eight samples (Skjoldal, 2021, see his Fig. S-1). Stage C5 also contributed to the small fraction since we assumed that 15% of C5 *C. finmarchicus* would be separated in this fraction (Skjoldal, 2021).

The calculated biomass of copepods in the small fraction is smaller than the recorded biomass (by 15–39%; Fig. 5), which allows for biomass of other groups. Small invertebrate larvae (e.g. gastropods, bivalves, echinoderms) and appendicularians are two groups that can be numerically abundant and distributed mainly in the small fraction (Skjoldal, 2021). We have not attempted to measure their size and estimate the biomass of these groups.

The biomass ratio of small to large copepods was remarkably similar across the three water masses. Thus, the ratio of small copepod species (dominated by *Oithona*) to *Calanus* varied from 0.12 to 0.2, being lowest in the Atlantic water where biomass of *Calanus* was highest (Fig. 1C). Reducing the estimated biomass of *C. finmarchicus* by 30% increases the ratio to 0.15 for the Atlantic water, closer to the ratios in mixed and Arctic waters. The Atlantic and Arctic waters represent two different domains in the biogeographical transition from boreal to Arctic conditions. This is reflected in the shift of dominance from *C. finmarchicus* to *C. glacialis* (Fig. 1D). We have not resolved the species within the genera of small copepods and can therefore not examine any changes in species composition between the two domains. However, we note a change of increased abundance and biomass of *Pseudocalanus* spp. in Arctic water and a corresponding decrease of other small copepods with boreal distributions (*Acartia, Centropages, Temora*) (Fig. 1).

The low and stable biomass ratio of small copepods to *Calanus* of ~0.15–0.2 reflects the strong dominance of the two *Calanus* species in each of their domains. The mechanisms behind this dominance are not clear but could involve predation control by feeding on eggs and/or nauplii of the small copepod species by the larger filter feeding *Calanus* copepods. Such mechanisms have been suggested for interactions among *Calanus* species including cannibalism (Bonnet *et al.*, 2004; Melle *et al.*, 2014; Frank-Gopolos *et al.*, 2017). The small copepod species are eaten by a wide range of predators including larval fish and many other zooplankton, and predation mortality is considered an important aspect of their population dynamics (Turner, 2004).

### Shift from *Calanus* to smaller zooplankton

The *Calanus* species are strongly dominant in terms of mesozooplankton biomass in the Barents Sea (Blachowiak-Samolyk *et al.*, 2008; Aarflot *et al.*, 2018), as we have also shown here (Figs 3 and 5). The strict and similar relationships between biomass of *Calanus* and biomass of the medium size fraction versus total biomass of zooplankton (Fig. 8) are interpreted to reflect that variation in total biomass is driven by variation in *Calanus* (Aarflot *et al.*, 2018). The variation in total biomass (and *Calanus*) is, in turn, driven by variable predation from the large and fluctuating Barents Sea capelin stock, combined with influence of warming and climate change (Stige *et al.*, 2014;Skjoldal *et al.*, 2022 ; Skjoldal, 2023).

Associated with a shift to lower biomass with increased predation from capelin, there is a shift in dominance from the medium to the small size fraction (Skjoldal *et al.*, 2022; Skjoldal, 2023). This is interpreted to reflect a decrease in the relative importance of *Calanus*, with a shift to smaller plankton forms as the overall biomass decreases. Our results for biomass of small copepods versus *Calanus* agree with this interpretation. The regression slope for small copepods is <1 and very similar to that for the small size fraction (Fig. 8). Thus, the biomass ratio of small copepod species to *Calanus* increases with decreasing total biomass (Fig. 7), consistent with expectations from increased predation by planktivorous fish like capelin that would select larger prey including *Calanus* species.

### Cladocerans play a small role in the Barents Sea

The estimated biomass of the two cladoceran taxa was very low (0.01 and 0.002 g dw m^−2^ in Atlantic water), being three orders of magnitude lower than the biomass of copepods. Cladocerans are primarily a freshwater group of plankton but are common also in brackish and estuarine waters. Only 8 species (out of a total of ~600 species) are found in neritic and marine waters, including two *Evadne* and two *Podon* species (Egloff *et al.*, 1997). These two taxa are common in the Baltic Sea and in coastal waters along Norway where the Norwegian Coastal Current flows as an extension of the outflow from the Baltic (Sætre, 2007). Our results demonstrate that species of *Evadne* and *Podon* are rare in the offshore waters of the Barents Sea, where they evidently play a very low role in the ecosystem.

## CONCLUSIONS

The zooplankton biomass in the Barents Sea is dominated by *Calanus* species, which make up ~85% of the estimated biomass of copepods and drive the overall variation in total zooplankton biomass. The second most important copepod taxon is *Oithona* spp., which make up on average ~10% of the biomass of copepods, followed by *Metridia* spp. (2–3%) and *Pseudocalanus* spp. (1–5%). The estimated biomass of cladocerans (*Evadne* and *Podon*) is three orders of magnitude lower than the biomass of copepods.

Zooplankton biomass has been monitored as dry weight in three size fractions on large-scale autumn surveys since the mid-1980s. The older copepodite stages (C4–6) of *Calanus* spp. are the main constituent of the medium size fraction (1–2 mm), while the young copepodite stages (C1–3) of *Calanus* along with other small copepods (*Oithona, Pseudocalanus, Microcalanus* and others) make up much of the biomass of the small fraction (<1 mm). The biomass ratio between small copepod species and *Calanus* spp. is similar (~0.15–0.2) across the biogeographical gradient from Atlantic (boreal) to Arctic waters, possibly reflecting predation control by the larger *Calanus* species on the smaller copepod species. The ratio of small copepods to *Calanus* shows a trend of increase with decreasing total zooplankton biomass, reflecting a shift from dominance of *Calanus* to a larger relative role of small copepod species at low biomass levels.

## Supplementary Material

Supplementary_revised_180923_fbad043

## Data Availability

The primary data for this paper is stored in the database at the Institute of Marine Research in Norway, Norwegian Marine Data Centre.

## References

[ref1] Aarflot, J. M., Hjøllo, S. S., Strand, E. and Skogen, M. D. (2022) Transportation and predation control structures the distribution of a key calanoid in the Nordic seas. Prog. Oceanogr., 202, 1–15, 102761. 10.1016/j.pocean.2022.102761.

[ref2] Aarflot, J. M., Skjoldal, H. R., Dalpadado, P. and Skern-Mauritzen, M. (2018) Contribution of *Calanus* species to the mesozooplankton biomass in the Barents Sea. ICES J. Mar. Sci., 75, 2342–2354. 10.1093/icesjms/fsx221.

[ref3] Bakke, J. L. (1977) Ecological studies on the deep-water pelagic community of Korsfjorden, western Norway: population dynamics of *Euchaeta norvegica* (Crustacea, Copepoda) from 1971 to 1974. Sarsia, 63, 49–55. 10.1080/00364827.1977.10411321.

[ref4] Båmstedt, U. and Matthews, J. B. L. (1975) Studies on the deep-water pelagic community of Korsfjorden, western Norway. The weight and biochemical composition of *Euchaeta norvegica* in relation to its life cycle. In Barnes, H. (ed.), Proc. 9^th^ Europ. Mar. Biol. Symp. 1975, Aberdeen University Press, Aberdeen, UK, pp. 311–327.

[ref5] Beaugrand, G., Lindley, J. A., Helaouet, P. and Bonnet, D. (2007) Macroecological study of *Centropages typicus* in the North Atlantic Ocean. Prog. Oceanogr., 72, 259–273. 10.1016/j.pocean.2007.01.002.

[ref6] Blachowiak-Samolyk, K., Søreide, J. E., Kwasniewski, S., Sundfjord, A., Hop, H., Falk-Petersen, S. and Hegseth, E. N. (2008) Hydrodynamic control of mesozooplankton abundance and biomass in northern Svalbard waters (79–81 N). Deep-Sea Res. II Top. Stud. Oceanogr., 55, 2210–2224. 10.1016/j.dsr2.2008.05.018.

[ref7] Bonnet, D., Titelman, J. and Harris, R. (2004) *Calanus* the cannibal. J. Plankton Res., 26, 937–948. 10.1093/plankt/fbh087.

[ref8] Böttger-Schnack, R. (1999) Taxonomy of Oncaeidae (Copepoda, Poecilostomatoida) from the Red Sea. – I. 11 species of *Triconia* gen. nov. and a redescription of *T. similis* (Sars) from Norwegian waters. Mitteil. Hamburg Zool. Museum und Institut, Hamburg, 96, 37–128.

[ref9] Brandão, M. C., Benedetti, F., Martini, S., Soviadan, Y. D., Irisson, J. O., Romagnan, J. B., Elineau, A., Desnos, C. et al. (2021) Macroscale patterns of oceanic zooplankton composition and size structure. Sci. Rep., 11, 1–19. 10.1038/s41598-021-94615-5. 34344925 PMC8333327

[ref10] Brooks, J. L. and Dodson, S. I. (1965) Predation, body size and composition of plankton. Science, 150, 28–35. 10.1126/science.150.3692.28. 17829740

[ref11] Castellani, C., Irigoien, X., Harris, R. P. and Holliday, N. P. (2007) Regional and temporal variation of *Oithona* spp. biomass, stage structure and productivity in the Irminger Sea, North Atlantic. J. Plankton Res., 29, 1051–1070. 10.1093/plankt/fbm079.

[ref12] Conover, R. J. (1988) Comparative life histories in the genera *Calanus* and *Neocalanus* in high latitudes of the northern hemisphere. Hydrobiol., 167-168, 127–142. 10.1007/BF00026299.

[ref13] Corkett, C. J. and McLaren, I. A. (1979) The biology of *Pseudocalanus*. Adv. Mar. Biol., 15, 1–231. 10.1016/S0065-2881(08)60404-6.

[ref14] Dalpadado, P., Arrigo, K. R., van Dijken, G. L., Skjoldal, H. R., Bagøien, E., Dolgov, A. V., Prokopchuk, I. P. and Sperfeld, E. (2020) Climate effects on temporal and spatial dynamics of phytoplankton and zooplankton in the Barents Sea. Prog. Oceanogr., 185, 21, 102320. 10.1016/j.pocean.2020.102320.

[ref15] Dvoretsky, V. G. and Dvoretsky, A. G. (2009) Spatial variations in reproductive characteristics of the small copepod *Oithona similis* in the Barents Sea. Mar. Ecol. Prog. Ser., 386, 133–146. 10.3354/meps08085.

[ref16] Dvoretsky, V. G. and Dvoretsky, A. G. (2015) Interannual variations in abundance and biomass of planktonic copepods *Oithona* in the Barents Sea. Biol. Bull., 42, 449–457. 10.1134/S1062359015050052. 26638241

[ref17] Dvoretsky, V. G. and Dvoretsky, A. G. (2023) Copepod assemblages in a large Arctic coastal area: a baseline summer study. Diversity, 15, 81. 10.3390/d15010081.

[ref18] Egloff, D. A., Fofonoff, P. W. and Onbé, T. (1997) Reproductive biology of marine cladocerans. Adv. Mar. Biol., 31, 79–167. 10.1016/S0065-2881(08)60222-9.

[ref19] Eriksen, E., Gjøsæter, H., Prozorkevich, D., Shamray, E., Dolgov, A., Skern-Mauritzen, M., Stiansen, J. E., Kovalev, Y. et al. (2018) From single species surveys towards monitoring of the Barents Sea ecosystem. Prog. Oceanogr., 166, 4–14. 10.1016/j.pocean.2017.09.007.

[ref20] Falk-Petersen, S., Mayzaud, P., Kattner, G. and Sargent, J. R. (2009) Lipids and life strategy of Arctic *Calanus*. Mar. Biol. Res., 5, 18–39. 10.1080/17451000802512267.

[ref21] Falk-Petersen, S., Pedersen, G., Kwasniewski, S., Hegseth, E. N. and Hop, H. (1999) Spatial distribution and life-cycle timing of zooplankton in the marginal ice zone of the Barents Sea during the summer melt season in 1995. J. Plankton Res., 21, 1249–1264. 10.1093/plankt/21.7.1249.

[ref22] Frank-Gopolos, T., Friis Møller, E. and Gissel Nielsen, T. (2017) The role of egg cannibalism for the *Calanus* succession in the Disko Bay, Western Greenland. Limnol. Oceanogr., 6, 865–883. 10.1002/lno.10472.

[ref23] Frost, B. W. (1989) A taxonomy of the marine calanoid copepod genus *Pseudocalanus*. Can. J. Zool., 67, 525–551. 10.1139/z89-077.

[ref24] Gallienne, C. P. and Robins, D. B. (2001) Is *Oithona* the most important copepod in the world's oceans? J. Plankton Res., 23, 1421–1432. 10.1093/plankt/23.12.1421.

[ref25] Grønvik, S. and Hopkins, C. C. E. (1984) Ecological investigations of the zooplankton community of Balsfjorden, northern Norway: generation cycle, seasonal vertical distribution, and seasonal variations in body weight and carbon and nitrogen content of the copepod *Metridia longa* (Lubbock). J. Exp. Mar. Biol. Ecol., 80, 93–107. 10.1016/0022-0981(84)90096-0.

[ref26] Hall, D. J., Threlkeld, S. T., Burns, C. W. and Crowley, P. H. (1976) The size-efficiency hypothesis and the size structure of zooplankton communities. Ann. Rev. Ecol. Syst., 7, 177–208. 10.1146/annurev.es.07.110176.001141.

[ref27] Halliday, N. C. (2001) A comparison of morphometric and geometric methods for the estimation of individual zooplankton volumes. Sarsia, 86, 101–105. 10.1080/00364827.2001.10420466.

[ref28] Hassel, A. (1986) Seasonal changes in zooplankton composition in the Barents Sea, with special attention to *Calanus* spp. (Copepoda). J. Plankton Res., 8, 329–339. 10.1093/plankt/8.2.329.

[ref29] Hassel, A., Endresen, B., Martinussen, M. B., Gjertsen, K., Knutsen, T. and Johannessen, M. E. (2020) Håndbok for Forskningsgruppe Plankton. Prøvetaking og analyse. Prosedyrer for prøvetaking og pre-analyse av dyre- og planteplankton på forskningsfartøy og i laboratorium på land, Version 6.0, Institute of Marine Research, Bergen, Norway, p. 173 (in Norwegian).

[ref30] Hay, S. J., Kiørboe, T. and Matthews, A. (1991) Zooplankton biomass and production in the North Sea during the autumn circulation experiment, October 1987-March 1988. Cont. Shelf Res., 11, 1453–1476. 10.1016/0278-4343(91)90021-W.

[ref31] Hernroth, L. (1985) Recommendations on Methodology for Marine Biological Studies in the Baltic Sea. Mesozooplankton Biomass Assessment, In The Baltic Marine Biologists Publication No. 10, pp. 6–9.

[ref32] Hirche, H. J. and Mumm, N. (1992) Distribution of dominant copepods in the Nansen Basin, Arctic Ocean, in summer. Deep-Sea Res., 39, 485–505. 10.1016/S0198-0149(06)80017-8.

[ref33] Ingvaldsen, R. B., Assmann, K. M., Primicerio, R., Fossheim, M., Polyakov, I. V. and Dolgov, A. V. (2021) Physical manifestations and ecological implications of Arctic Atlantification. Nature Rev. Earth Env., 2, 874–889. 10.1038/s43017-021-00228-x.

[ref34] Klein Breteler, W. C. M., Fransz, H. G. and Gonzalez, S. R. (1982) Growth and development of four calanoid copepod species under experimental and natural conditions. Neth. J. Sea Res., 16, 195–207. 10.1016/0077-7579(82)90030-8.

[ref35] Kosobokova, K. N. and Hirche, H.-J. (2009) Biomass of zooplankton in the eastern Arctic Ocean – a base line study. Prog. Oceanogr., 82, 265–280. 10.1016/j.pocean.2009.07.006.

[ref36] Langbehn, T. J., Aarflot, J. M., Freer, J. and Varpe, Ø. (2023) Visual predation risk and spatial distributions of large Arctic copepods along gradients of sea ice and bottom depth. Limnol. Oceanogr., 68, 1388–1405. 10.1002/lno.12354.

[ref37] Lenz, J. (2000) Introduction. In Harris, R., Wiebe, P., Lenz, J., Skjoldal, H. R. and Huntley, M. (eds.), ICES Zooplankton Methodology Manual, Academic Press, New York, pp. 1–32.

[ref38] Longhurst, A. R. (1985) The structure and evolution of plankton communities. Prog. Oceanogr., 15, 1–35. 10.1016/0079-6611(85)90036-9.

[ref39] Marshall, S. M., Nicholls, A. G. and Orr, A. P. (1934) On the biology of *Calanus finmarchicus*. 5. Seasonal distribution, size, weight and chemical composition in Loch Striven in 1933, and their relation to phytoplankton. J. Mar. Biol. Ass. UK, 19, 793–827. 10.1017/S0025315400046804.

[ref40] Mauchline, J. (1995) Bathymetric adaptations of life history patterns of congeneric species (Euchaeta: Calanoida) in a 2000 m water column. ICES J. Mar. Sci., 52, 511–516. 10.1016/1054-3139(95)80064-6.

[ref41] McLaren, I. A., Head, E. and Sameoto, D. D. (2001) Life cycles and seasonal distributions of *Calanus finmarchicus* on the central Scotian shelf. Can. J. Fish. Aquat. Sci., 58, 659–670. 10.1139/f01-007.

[ref42] McLaren, I. A., Sevigny, J.-M. and Corkett, C. J. (1989) Temperature-dependent development in *Pseudocalanus* species. Can. J. Zool., 67, 559–564. 10.1139/z89-079.

[ref43] Melle, W., Runge, J. A., Head, E., Plourde, S., Castellani, C., Licandro, P., Jónasdóttir, S. H., Johnson, C. et al. (2014) The North Atlantic Ocean as habitat for *Calanus finmarchicus*: environmental factors and life history traits. Prog. Oceanogr., 129, 244–284. 10.1016/j.pocean.2014.04.026.

[ref44] Melle, W. and Skjoldal, H. R. (1998) Reproduction and development of *Calanus finmarchicus*, *C. glacialis* and *C. hyperboreus* in the Barents Sea. Mar. Ecol. Prog. Ser., 169, 211–228. 10.3354/meps169211.

[ref45] Möllmann, C., Köster, F., Kornilovs, G. and Sidrevics, L. (2002) Long-term trends in abundance of cladocerans in the Central Baltic Sea. Mar. Biol., 141, 343–352.

[ref46] Nichols, J. H. and Thompson, A. B. (1991) Mesh selection of copepodite and nauplius stages of four calanoid copepod species. J. Plankton Res., 13, 661–671. 10.1093/plankt/13.3.661.

[ref47] Norrbin, M. F. (1991) Gonad maturation as an indication of seasonal cycles for several species of small copepods in the Barents Sea. Polar Res., 10, 205–211.

[ref48] Norrbin, M. F. (1994) Seasonal patterns in gonad maturation, sex ratio and size in some small, high-latitude copepods: implications for overwintering tactics. J. Plankton Res., 16, 115–131. 10.1093/plankt/16.2.115.

[ref49] Paffenhöfer, G. A. (1993) On the ecology of marine cyclopoid copepods (Crustacea, Copepoda). J. Plankton Res., 15, 37–55. 10.1093/plankt/15.1.37.

[ref50] Pasternak, A., Arashkevich, E., Reigstad, M., Wassmann, P. and Falk-Petersen, S. (2008) Dividing mesozooplankton into upper and lower size groups: applications to the grazing impact in the marginal ice zone of the Barents Sea. Deep-Sea Res. II Top. Stud. Oceanogr., 55, 2245–2256. 10.1016/j.dsr2.2008.05.002.

[ref51] Pearre, S. Jr. (1980) The copepod width–weight relation and its utility in food chain research. Can. J. Zool., 58, 1884–1891. 10.1139/z80-257.

[ref52] R Core Team (2016) R: A Language and Environment for Statistical Computing. R Foundation for Statistical Computing, Vienna, Austria. https://www.R-project.org/.

[ref53] Reigstad, M., Carroll, J., Slagstad, D., Ellingsen, I. and Wassmann, P. (2011) Intra-regional comparison of productivity, carbon flux and ecosystem composition within the northern Barents Sea. Prog. Oceanogr., 90, 33–46. 10.1016/j.pocean.2011.02.005.

[ref54] Sabatini, M. and Kiørboe, T. (1994) Egg production, growth and development of the cyclopoid copepod *Oithona similis*. J. Plankton Res., 16, 1329–1351. 10.1093/plankt/16.10.1329.

[ref55] Sætre, R. (2007) The Norwegian Coastal Current – Oceanography and Climate, Tapir Academic Press, Trondheim, p. 159.

[ref56] Sameoto, D., Wiebe, P., Runge, J., Postel, L., Dunn, J., Miller, C. and Coombs, S. (2000) Collecting zooplankton. In Harris, R., Wiebe, P., Lenz, J., Skjoldal, H. R. and Huntley, M. (eds.), ICES Zooplankton Methodology Manual, Academic Press, New York, pp. 55–81.

[ref57] Skagseth, Ø., Drinkwater, K. F. and Terrile, E. (2011) Wind- and buoyancy-induced transport of the Norwegian coastal current in the Barents Sea. J. Geophys. Res. Oceans, 116, 15. 10.1029/2011JC006996.

[ref58] Skjoldal, H. R. (2021) Species composition of three size fractions of zooplankton used in routine monitoring of the Barents Sea ecosystem. J. Plankton Res., 43, 762–772. 10.1093/plankt/fbab056. 34584493 PMC8461643

[ref59] Skjoldal, H. R. (2022) Ecosystem assessment of the Central Arctic Ocean: description of the ecosystem. ICES Coop. Res. Rep., 355, 341. 10.17895/ices.pub.20191787.

[ref60] Skjoldal, H. R. (2023) Size-fractioned zooplankton biomass in the Barents Sea ecosystem: changes during four decades of warming and four capelin collapses (1980-2020). ICES J. Mar. Sci., 1–14. 10.1093/icesjms/fsad057.

[ref61] Skjoldal, H. R., Aarflot, J. M., Bagøien, E., Skagseth, Ø., Rønning, J. and Lien, V. S. (2021) Seasonal and interannual variability in abundance and population development of *Calanus finmarchicus* at the western entrance to the Barents Sea, 1995–2019. Prog. Oceanogr., 195, 20, 102574. 10.1016/j.pocean.2021.102574.

[ref62] Skjoldal, H. R., Eriksen, E. and Gjøsæter, H. (2022) Size-fractioned zooplankton biomass in the Barents Sea: spatial patterns and temporal variations during three decades of warming and strong fluctuations of the capelin stock (1989–2020). Prog. Oceanogr., 206, 18, 102852. 10.1016/j.pocean.2022.102852.

[ref63] Skjoldal, H. R., Hassel, A., Rey, F. and Loeng, H. (1987) Spring phytoplankton development and zooplankton reproduction in the central Barents Sea in the period 1979–1984. In Loeng, H. (ed.), The Effect of Oceanographic Conditions on Distribution and Population Dynamics of Commercial Fish Stocks in the Barents Sea, Proceedings of the Third Soviet–Norwegian Symposium, Murmansk, 26–28 May 1986. Institute of Marine Research, Bergen, Norway, pp. 59–89.

[ref64] Skjoldal, H. R., Prokopchuk, I., Bagøien, E., Dalpadado, P., Nesterova, V., Rønning, J. and Knutsen, T. (2019) Comparison of Juday and WP2 nets used in joint Norwegian–Russian monitoring of zooplankton in the Barents Sea. J. Plankton Res., 41, 759–769. 10.1093/plankt/fbz054.

[ref65] Skjoldal, H. R. and Rey, F. (1989) Pelagic production and variability of the Barents Sea ecosystem. In Sherman, K. and Alexander, L. M. (eds.), Biomass Yields and Geography of Large Marine Ecosystems. AAAS Selected Symposium, 111, Westview Press, Inc., Colorado, USA, pp. 241–286.

[ref66] Skjoldal, H. R., Wiebe, P. H., Postel, L., Knutsen, T., Kaartvedt, S. and Sameoto, D. D. (2013) Intercomparison of zooplankton (net) sampling systems: results from the ICES/GLOBEC sea-going workshop. Prog. Oceanogr., 108, 1–42. 10.1016/j.pocean.2012.10.006.

[ref67] Smith, P. E., Counts, R. C. and Clutter, R. I. (1968) Changes in filtering efficiency of plankton nets due to clogging under tow. J. Cons. perm. int. Explor. Mer, 32, 232–248. 10.1093/icesjms/32.2.232.

[ref68] Stige, L. C., Dalpadado, P., Orlova, E., Boulay, A.-C., Durant, J. M., Ottersen, G. and Stenseth, N. C. (2014) Spatiotemporal statistical analyses reveal predator-driven zooplankton fluctuations in the Barents Sea. Prog. Oceanogr., 120, 243–253. 10.1016/j.pocean.2013.09.006.

[ref69] Strand, E., Bagøien, E., Edwards, M., Broms, C. and Klevjer, T. (2020) Spatial distributions and seasonality of four *Calanus* species in the Northeast Atlantic. Prog. Oceanogr., 185, 18, 102344. 10.1016/j.pocean.2020.102344.

[ref70] Tande, K. (1991) *Calanus* in North Norwegian fjords and in the Barents Sea. Polar Res., 10, 389–408. 10.1111/j.1751-8369.1991.tb00661.x.

[ref71] Tande, K. S. (1982) Ecological investigations on the zooplankton community of Balsfjorden, northern Norway: generation cycles, and variation in body weight and body content of carbon and nitrogen related to overwintering and reproduction in the copepod *Calanus finmarchicus* (Gunnerus). J. Exp. Mar. Biol. Ecol., 62, 129–142. 10.1016/0022-0981(82)90087-9.

[ref72] Turner, J. T. (2004) The importance of small planktonic copepods and their roles in pelagic marine food webs. Zool. Stud., 43, 255–266.

[ref73] Wassmann, P., Vernet, M., Mitchell, G. and Rey, F. (1990) Mass sedimentation of *Phaeocystis pouchetii* in the Barents Sea. Mar. Ecol. Prog. Ser., 66, 183–195. 10.3354/meps066183.

[ref74] WGIBAR (2021) Working group on the integrated assessments of the Barents Sea (WGIBAR). ICES Sci. Rep., 3, 236. 10.17895/ices.pub.8241.

[ref75] Wiborg, K. F. (1954) Investigations on zooplankton in coastal and offshore waters of western and northwestern Norway with special reference to the copepods. Fisk. dir. Skr. Ser. Havunders., 11, 1–245.

