# Supplementary material for: Abundance and biomass of copepods and cladocerans in Atlantic and Arctic domains of the Barents Sea ecosystem
Source: J Plankton Res. 2023 Oct 12;45(6):870–84. doi: 10.1093/plankt/fbad043 (PMC10710905; doi:10.1093/plankt/fbad043)
Supplement: Supplementary_revised_180923_fbad043 [file supplementary_revised_180923_fbad043.docx]

**Abundance and biomass of copepods and cladocerans in Atlantic and Arctic domains of the Barents Sea ecosystem –** Hein Rune Skjoldal and Johanna Myrseth Aarflot

**Supplementary material**

Table S-1. Species or genera of copepods and cladocerans counted in samples from the Barents Sea. Columns show copepodite stage number, individual weight used to estimate biomass from numbers, mean estimated dry weight biomass, and proportion of biomass assigned to the three size fractions.

| Species/genus | Stage | Weight (μg) ^1^ | Biomass (g dw m^-2^) | Proportion (%) ^2^ | | |
| --- | --- | --- | --- | --- | --- | --- |
|  |  |  |  | Small | Medium | Large |
| *Calanus finmarchicus* | C1 | 1.5 | 0.005 | 100 |  |  |
|  | C2 | 4 | 0.019 | 95 | 5 |  |
|  | C3 | 13 | 0.086 | 90 | 7 | 3 |
|  | C4 | 70 | 0.848 | 47 | 48 | 5 |
|  | C5 | 250 | 2.403 | 15 | 80 | 5 |
|  | C6 | 235 | 0.235 | 15 | 80 | 5 |
| *Calanus glacialis* | C1 | 8 | 0.003 | 95 | 5 |  |
|  | C2 | 16 | 0.003 | 90 | 10 |  |
|  | C3 | 40 | 0.023 | 85 | 13 | 2 |
|  | C4 | 185 | 0.128 | 30 | 66 | 4 |
|  | C5 | 600 | 0.231 |  | 90 | 10 |
|  | C6 | 810 | 0.084 |  | 90 | 10 |
| *Calanus hyperboreus* | C1 | 10 | 0.000 | 95 | 5 |  |
|  | C2 | 40 | 0.001 | 90 | 10 |  |
|  | C3 | 140 | 0.004 | 80 | 15 | 5 |
|  | C4 | 500 | 0.058 |  | 90 | 10 |
|  | C5 | 2000 | 0.064 |  | 67 | 33 |
|  | C6 | 3500 | 0.028 |  | 10 | 90 |
| *Metridia* spp. | C1-C3 | 15 | 0.013 | 90 | 7 | 3 |
|  | C4-C5 | 50 | 0.068 | 56 | 40 | 4 |
|  | C6 | 150 | 0.058 |  | 90 | 10 |
| *Pseudocalanus* spp. | C1-C3 | 3 | 0.016 | 98 | 2 |  |
|  | C4-C5 | 8 | 0.039 | 97 | 3 |  |
|  | C6 | 12 | 0.008 | 97 | 3 |  |
| *Pareuchaeta* spp. | C1-C3 | 52 | 0.002 |  | 97 | 3 |
|  | C4-C5 | 830 | 0.023 |  | 75 | 25 |
|  | C6 | 3000 | 0.006 |  |  | 100 |
| *Microcalanus* spp. | C1-6 | 1.5 | 0.042 | 100 |  |  |
| *Acartia* spp. | C1-6 | 10 | 0.029 | 100 |  |  |
| *Centropages* spp. | C1-6 | 20 | 0.006 | 100 |  |  |
| *Temora* spp. | C1-6 | 20 | 0.030 | 100 |  |  |
| *Oithona* spp. | C1-6 | 2 | 0.447 | 100 |  |  |
| *Oncaea* spp. | C1-6 | 4 | 0.013 | 100 |  |  |
| *Evadne* spp. |  | 7 | 0.006 | 100 |  |  |
| *Podon* spp. |  | 7 | 0.001 | 100 |  |  |

^1^ Weights of stages of *Calanus* species from Aarflot et al. (2018), based on sources given in that paper. Weights of other taxa are based on: *Metridia* – Grønvik and Hopkins (1984), Hirche and Mumm (1992), and Halliday (2001); *Pseudocalanus* – Corkett and McLaren (1979), Klein Breteler et al. (1982), and Hay et al. (1991); *Paraeuchaeta* – Båmstedt and Matthews (1975) and Bakke (1977); *Acartia*, *Centropages*, *Temora* – Klein Breteler et al. (1982) and Hay et al. (1991); *Oithona* – McLaren et al. (1989) and Hay et al. (1991); *Microcalanus*, *Oncaea/Triconia* – Hay et al. (1991) and Skjoldal et al. (2013); *Evadne*, *Podon* – Hernroth (1985) and Skjoldal et al. (2013).

^2^ Based on Skjoldal (2021).

Table S-2. Number of zooplankton sampling stations by year (1983-2016) in Arctic, mixed, and Atlantic water masses (defined by temperature at 50 m depth <0^o^C, 0-3^o^C, and >3^o^C, respectively; see Aarflot et al., 2018) during extended summer (May-September) and winter (October-April) periods. Month is given as number from 1 (January) to 12 (December).

|  | ‘Summer' |  |  |  | ‘Winter' |  |  |  |
| --- | --- | --- | --- | --- | --- | --- | --- | --- |
| Year | Month | Arctic | Mixed | Atlantic | Month | Arctic | Mixed | Atlantic |
| 1983 | 6 | 2 | 1 | 0 |  |  |  |  |
| 1984 | 5, 6 | 5 | 5 | 1 |  |  |  |  |
| 1987 | 6 | 0 | 0 | 1 |  |  |  |  |
| 1988 | 7 | 0 | 3 | 3 |  |  |  |  |
| 1991 | 9 | 1 | 1 | 0 |  |  |  |  |
| 1992 | 5, 6, 9 | 4 | 4 | 19 | 10 | 0 | 2 | 7 |
| 1995 | 6, 8 | 0 | 2 | 6 | 1, 3, 10 | 0 | 2 | 9 |
| 1996 | 5, 7, 8 | 0 | 5 | 7 | 1, 2, 3, 10 | 2 | 2 | 8 |
| 1997 | 6, 8, 9 | 0 | 3 | 9 | 2, 3 | 1 | 1 | 6 |
| 1998 | 6, 8 | 0 | 2 | 6 | 1, 3, 4, 10 | 0 | 3 | 12 |
| 1999 | 7, 8 | 0 | 1 | 7 | 1, 3, 4, 10 | 0 | 2 | 14 |
| 2000 | 8, 9 | 0 | 1 | 5 | 1, 3, 4, 10 | 0 | 2 | 12 |
| 2001 | 5, 8 | 0 | 2 | 6 | 1, 3, 10 | 0 | 3 | 9 |
| 2002 | 6, 8 | 0 | 2 | 6 | 1, 3, 4, 10 | 0 | 3 | 12 |
| 2003 | 6, 9 | 1 | 0 | 6 | 1, 3, 4, 10 | 1 | 3 | 15 |
| 2004 | 6, 8 | 0 | 1 | 15 | 1, 3, 4 | 0 | 3 | 19 |
| 2005 | 5, 6, 8, 9 | 5 | 5 | 12 | 1, 3, 10 | 0 | 1 | 9 |
| 2006 | 5, 6, 8 | 0 | 3 | 31 | 1, 3, 10 | 0 | 1 | 10 |
| 2007 | 5, 6, 8 | 0 | 0 | 13 | 1, 3, 4, 11 | 0 | 3 | 14 |
| 2008 | 6, 9 | 0 | 0 | 8 | 2, 3, 4 | 0 | 2 | 8 |
| 2009 | 6, 8 | 0 | 1 | 4 | 2, 3, 10 | 0 | 2 | 7 |
| 2010 | 6, 8, 9 | 0 | 7 | 7 | 1, 3, 10 | 0 | 2 | 9 |
| 2011 | 6, 8, 9 | 4 | 2 | 5 | 1, 3, 4, 10 | 0 | 4 | 9 |
| 2012 | 8, 9 | 1 | 4 | 4 | 3, 10 | 0 | 0 | 7 |
| 2013 | 8, 9 | 3 | 2 | 7 | 3, 4 | 0 | 0 | 8 |
| 2014 | 5, 6, 8 | 0 | 3 | 9 | 1, 3, 11 | 0 | 1 | 7 |
| 2015 | 5, 8 | 0 | 1 | 7 | 1, 3, 4, 11 | 1 | 0 | 15 |
| 2016 | 7, 8 | 0 | 0 | 4 | 1, 3, 4 | 0 | 1 | 11 |
| Sum | Summer' | 26 | 61 | 208 | Winter' | 5 | 43 | 237 |
| Total |  |  |  | 295 |  |  |  | 285 |

Table S-3. Frequency of occurrence (% of samples), abundance, and estimated biomass of copepod and cladoceran taxa. Mean, median, and maximum values of abundance and biomass are given for samples collected in Atlantic, mixed, and Arctic water masses in ‘summer’ (May-September) for samples collected between 1983 and 2016 (see Table S-2).

|  | **Presence** | **Abundance (# ind. m^-2^)** | | | **Biomass (g dw m^-2^)** | | |
| --- | --- | --- | --- | --- | --- | --- | --- |
| **Taxon** | % | **Mean** | **Median** | **Max** | **Mean** | **Median** | **Max** |
|  |  |  |  |  |  |  |  |
| Atlantic water, n = 208 | |  |  |  |  |  |  |
| *Calanus finmarchicus* | 100 | 77940 | 34304 | 988160 | 7.04 | 3.39 | 46.25 |
| *C. glacialis* | 43 | 1934 | 0 | 123392 | 0.42 | 0.00 | 13.68 |
| *C. hyperboreus* | 50 | 234 | 8 | 5376 | 0.18 | 0.01 | 3.07 |
| *Metridia* spp. | 88 | 3947 | 1472 | 48128 | 0.16 | 0.08 | 1.50 |
| *Paraeuchaeta* spp. | 29 | 109 | 0 | 8528 | 0.04 | 0.00 | 1.70 |
| *Pseudocalanus* spp. | 93 | 11511 | 3328 | 423936 | 0.07 | 0.02 | 1.93 |
| *Acartia* spp. | 26 | 5692 | 0 | 370176 | 0.06 | 0.00 | 3.70 |
| *Centropages* spp. | 6 | 241 | 0 | 13312 | 0.00 | 0.00 | 0.27 |
| *Microcalanus* spp. | 82 | 42936 | 13860 | 519552 | 0.06 | 0.02 | 0.78 |
| *Temora* spp. | 17 | 3233 | 0 | 270336 | 0.06 | 0.00 | 5.41 |
| *Oithona* spp. | 99 | 329400 | 184960 | 3928064 | 0.66 | 0.37 | 7.86 |
| *Oncaea* spp. | 63 | 5355 | 768 | 131072 | 0.02 | 0.00 | 0.52 |
| *Evadne* spp. | 11 | 2058 | 0 | 271872 | 0.01 | 0.00 | 1.90 |
| *Podon* spp. | 4 | 290 | 0 | 24576 | 0.00 | 0.00 | 0.17 |
|  |  |  |  |  |  |  |  |
| Mixed water mass, n = 61 | |  |  |  |  |  |  |
| *Calanus finmarchicus* | 100 | 19897 | 8848 | 326144 | 1.40 | 0.81 | 11.89 |
| *C. glacialis* | 90 | 7571 | 1536 | 47616 | 1.47 | 0.41 | 7.49 |
| *C. hyperboreus* | 67 | 563 | 64 | 6672 | 0.24 | 0.05 | 2.08 |
| *Metridia* spp. | 70 | 3373 | 320 | 57984 | 0.11 | 0.01 | 1.31 |
| *Paraeuchaeta* spp. | 15 | 28 | 0 | 928 | 0.01 | 0.00 | 0.43 |
| *Pseudocalanus* spp. | 100 | 32664 | 7168 | 303104 | 0.19 | 0.05 | 1.89 |
| *Acartia* spp. | 13 | 121 | 0 | 2048 | 0.00 | 0.00 | 0.02 |
| *Centropages* spp. | 5 | 2 | 0 | 48 | 0.00 | 0.00 | 0.00 |
| *Microcalanus* spp. | 74 | 15778 | 3840 | 147456 | 0.02 | 0.01 | 0.22 |
| *Temora* spp. | 7 | 84 | 0 | 2048 | 0.00 | 0.00 | 0.04 |
| *Oithona* spp. | 97 | 196537 | 79360 | 2248704 | 0.39 | 0.16 | 4.50 |
| *Oncaea* spp. | 62 | 3131 | 384 | 77312 | 0.01 | 0.00 | 0.31 |
| *Evadne* spp. | 5 | 205 | 0 | 10752 | 0.00 | 0.00 | 0.08 |
| *Podon* spp. | 0 | 0 | 0 | 0 | 0.00 | 0.00 | 0.00 |
|  |  |  |  |  |  |  |  |
| Arctic water, n = 26 |  |  |  |  |  |  |  |
| *Calanus finmarchicus* | 100 | 9535 | 5840 | 35584 | 0.53 | 0.29 | 2.26 |
| *C. glacialis* | 100 | 15099 | 6848 | 102896 | 2.19 | 1.15 | 9.89 |
| *C. hyperboreus* | 81 | 672 | 456 | 3024 | 0.47 | 0.24 | 2.51 |
| *Metridia* spp. | 69 | 4664 | 1408 | 28288 | 0.12 | 0.06 | 0.56 |
| *Paraeuchaeta* spp. | 8 | 1 | 0 | 16 | 0.00 | 0.00 | 0.03 |
| *Pseudocalanus* spp. | 100 | 22777 | 16768 | 69120 | 0.13 | 0.09 | 0.35 |
| *Acartia* spp. | 4 | 158 | 0 | 4096 | 0.00 | 0.00 | 0.04 |
| *Centropages* spp. | 4 | 20 | 0 | 512 | 0.00 | 0.00 | 0.01 |
| *Microcalanus* spp. | 54 | 18338 | 4032 | 110080 | 0.03 | 0.01 | 0.17 |
| *Temora* spp. | 4 | 10 | 0 | 256 | 0.00 | 0.00 | 0.01 |
| *Oithona* spp. | 81 | 210166 | 156160 | 718080 | 0.42 | 0.31 | 1.44 |
| *Oncaea* spp. | 42 | 5470 | 0 | 35712 | 0.02 | 0.00 | 0.14 |
| *Evadne* spp. | 0 | 0 | 0 | 0 | 0.00 | 0.00 | 0.00 |
| *Podon* spp. | 0 | 0 | 0 | 0 | 0.00 | 0.00 | 0.00 |

Table S-4. Measured biomass in 3 size fractions and total (sum of fractions) for the complementary half-samples to the extended summer (May-September) data set with species counts used to estimate biomass of copepods and cladocerans. Values are given as mean, median, and standard deviation (SD) for set of samples with n = 208, 61, and 26 sampling stations for Atlantic, Mixed, and Arctic waters, respectively.

| Fraction |  | Biomass (g dw m^-2^) | | |
| --- | --- | --- | --- | --- |
|  |  | Atlantic | Mixed | Arctic |
| Large | Mean | 0.95 | 0.75 | 2.67 |
|  | Median | 0.41 | 0.18 | 1.94 |
|  | SD | 1.37 | 1.46 | 2.93 |
| Medium | Mean | 4.06 | 3.17 | 4.09 |
|  | Median | 2.72 | 1.31 | 2.34 |
|  | SD | 4.40 | 3.92 | 4.66 |
| Small | Mean | 3.72 | 2.19 | 1.42 |
|  | Median | 2.31 | 1.43 | 1.26 |
|  | SD | 4.10 | 2.12 | 0.81 |
| Total | Mean | 8.73 | 6.11 | 8.18 |
|  | Median | 6.14 | 4.16 | 5.25 |
|  | SD | 7.65 | 5.59 | 7.18 |


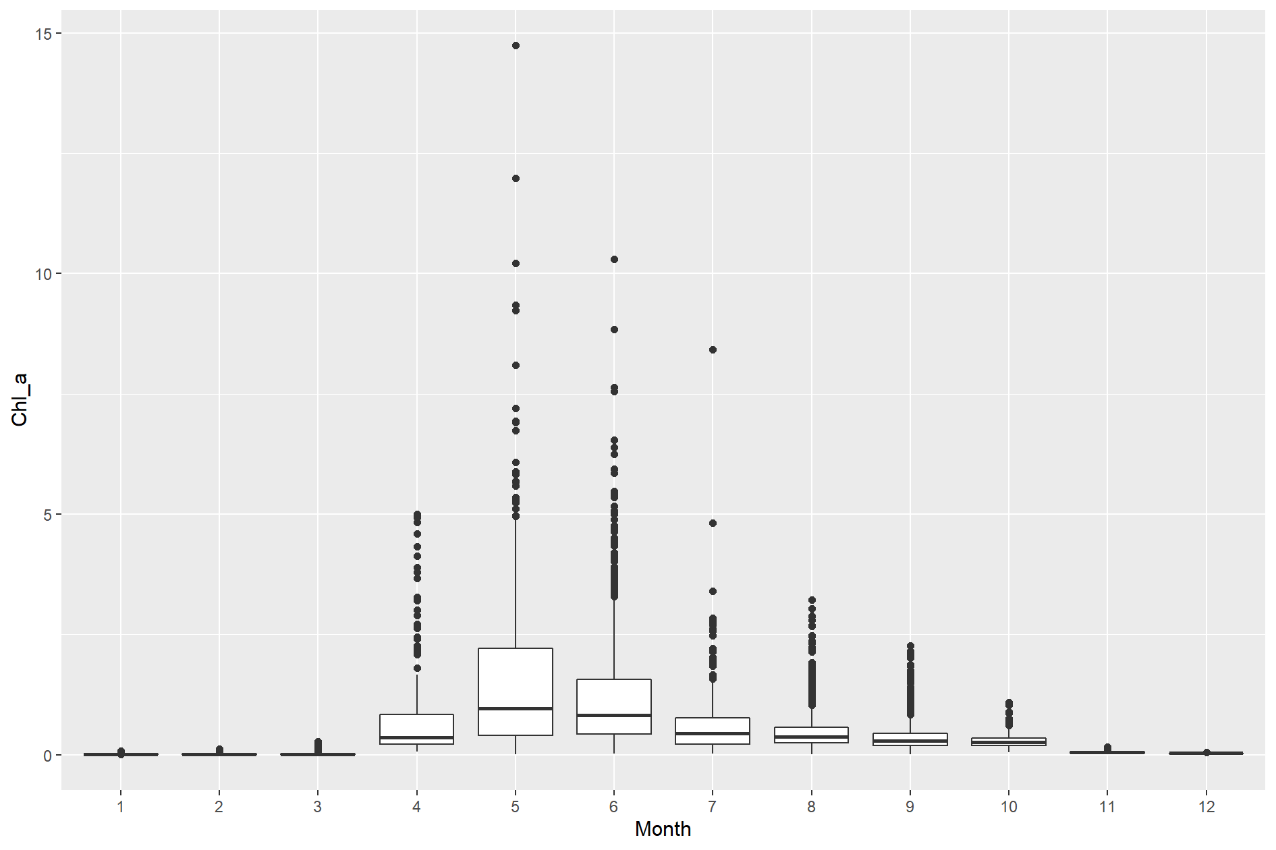


Fig. S-1. Box-whisker plots of concentration of chlorophyll *a* in the Barents Sea by month from January to December. The data are from the IMR database for 1980-2017 with a total of 61,702 samples from discrete depths at 8,074 oceanographic stations with wide spatial coverage. The values are averages over the sampling depths at stations, and plots show median (horizontal line), 25 and 75 percentiles (box), 5 and 95 percentiles (vertical line), and statistical outliers (dots).


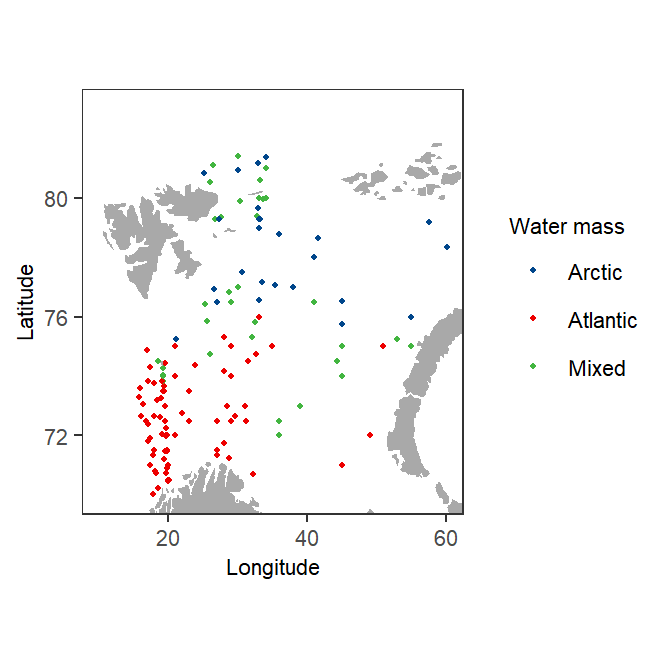


Fig. S-2. Distribution of sampling stations in the Barents Sea with species counts and estimated biomass of copepods and cladocerans. The stations have been classified according to water mass identified by colored symbols. Arctic, mixed, and Atlantic water masses are defined by temperature at 50 m depth <0^o^C, 0-3^o^C, and >3^o^C, respectively (see Aarflot et al., 2018).


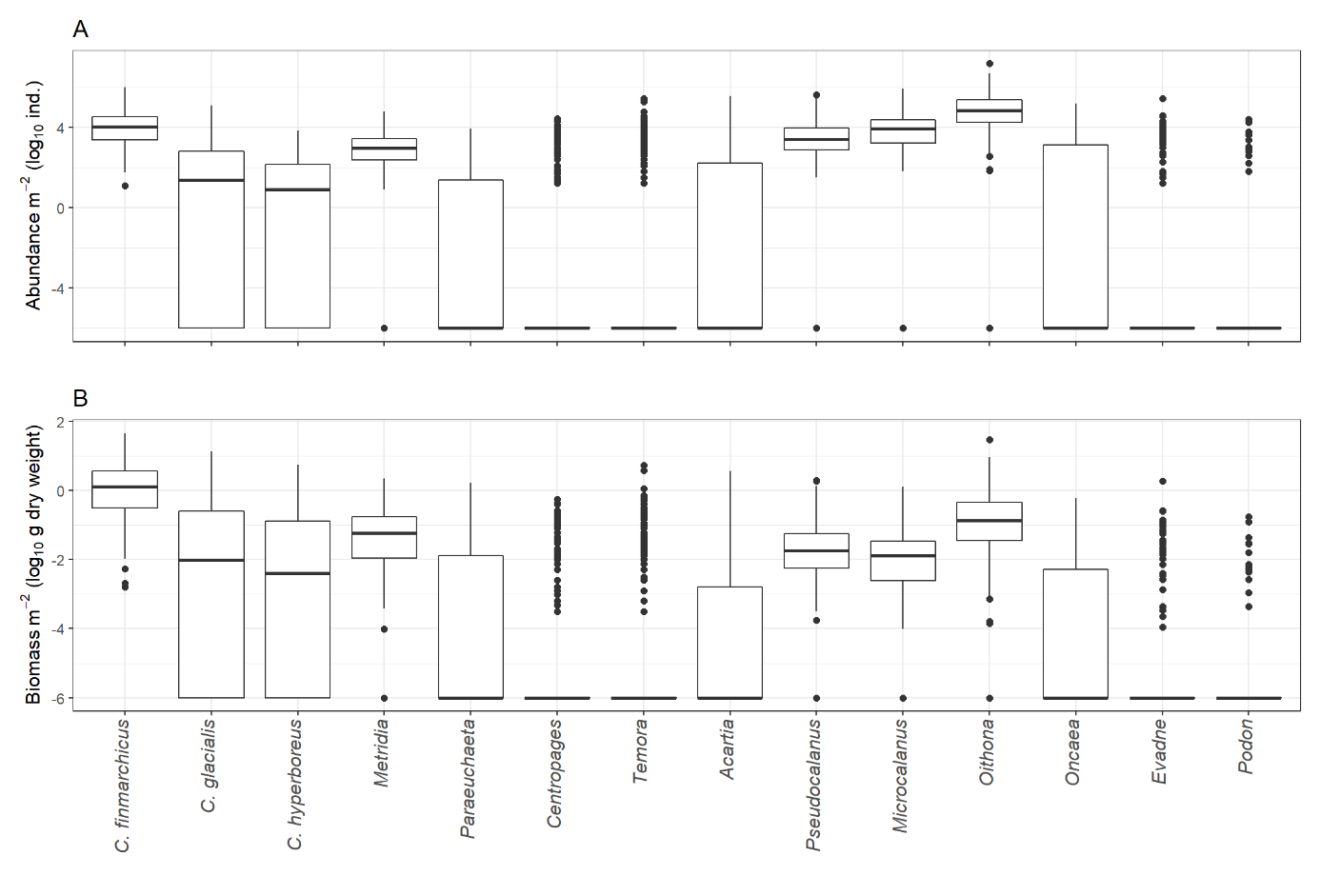


Fig. S-3. Box-whisker diagrams of (A) abundance and (B) calculated biomass of 12 copepod and 2 cladoceran taxa for the total data set (n = 580 stations). Data are log10 transformed, with a low value of 10^-6^ added to abundance and biomass data; the log10 value of -6 is therefore equivalent to 0 (zero). The horizontal bars, boxes, whiskers, and individual points are median values, 25-75 percentiles, 5-95 percentiles, and ‘outliers’, respectively.


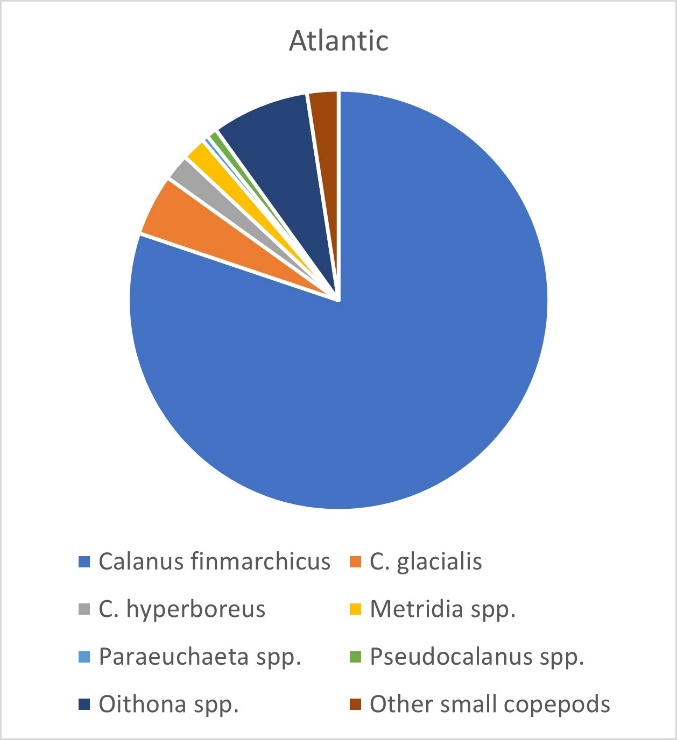

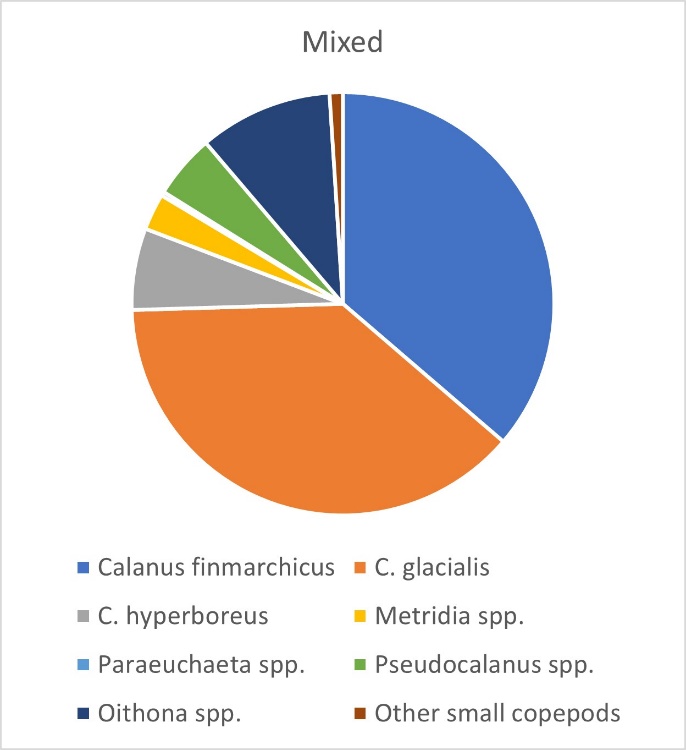

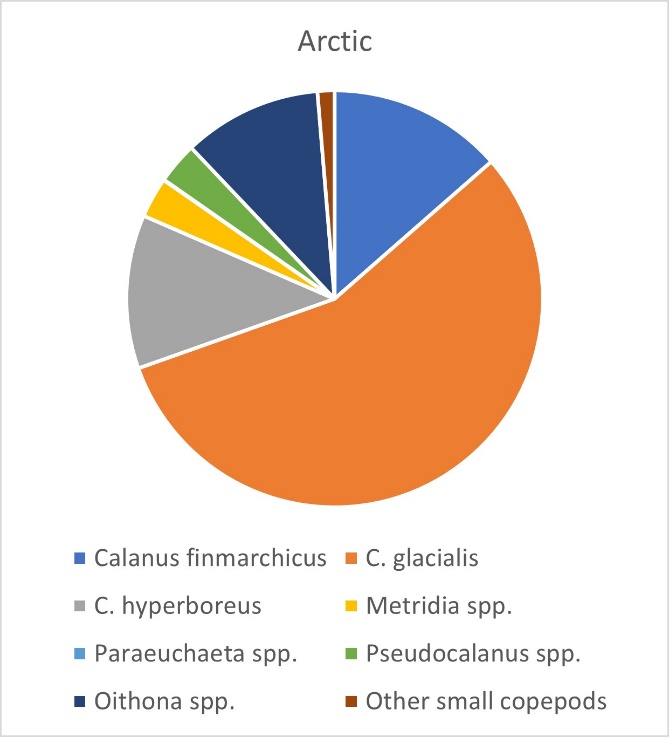


Fig. S-4. Pie-chart diagrams of relative contribution by copepod taxa to estimated total biomass of copepods for samples from Atlantic, Mixed, and Arctic water masses for the extended summer (May-September) data set (n = 208, 61, and 26, respectively). The total estimated biomass of copepods was 8.8, 3.9, and 3.9 g dw m^-2^ for the Atlantic, Mixed, and Arctic water masses, respectively.


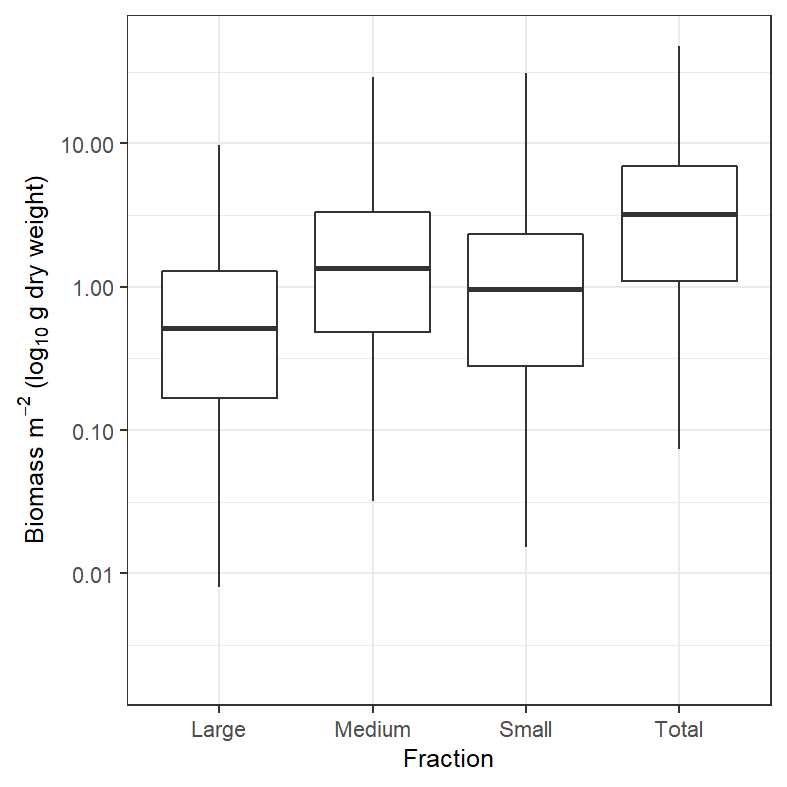


Fig. S-5. Box-whisker diagrams of biomass of the large, medium, and small size fractions, and of total zooplankton biomass (sum of the 3 fractions), for the complementary set of half-samples with biomass determination to the set of half-samples with species counts used in this study. Results for the total data set (n = 580).


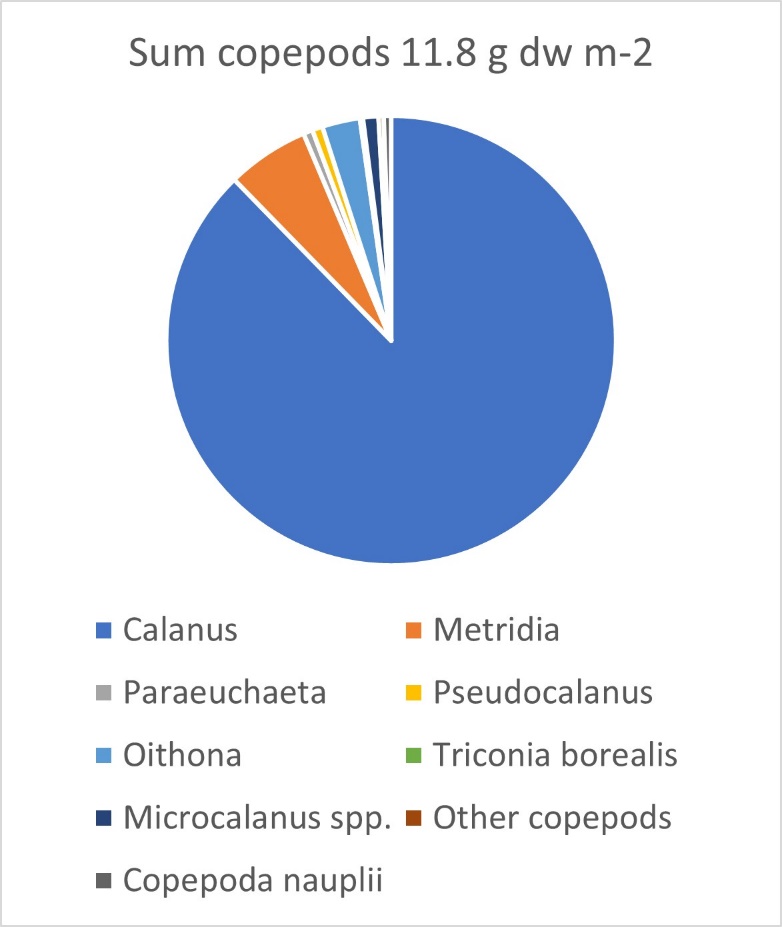


Fig. S-6. Pie-chart diagram of relative contribution by copepod taxa to estimated total biomass of copepods reported by Blachowiak-Samolyk (2008); based on data in their Table 3.
